# A paradoxical knowledge gap in science for critically endangered fishes and game fishes during the sixth mass extinction

**DOI:** 10.1038/s41598-021-87871-y

**Published:** 2021-04-19

**Authors:** Christopher S. Guy, Tanner L. Cox, Jacob R. Williams, Colter D. Brown, Robert W. Eckelbecker, Hayley C. Glassic, Madeline C. Lewis, Paige A. C. Maskill, Lauren M. McGarvey, Michael J. Siemiantkowski

**Affiliations:** 1 U.S. Geological Survey, Montana Cooperative Fishery Research Unit, Department of Ecology, Montana State University, PO Box 173460, Bozeman, MT 59717 USA; 2 Montana Cooperative Fishery Research Unit, Department of Ecology, Montana State University, PO Box 173460, Bozeman, MT 59717 USA; 3 Montana Fish, Wildlife and Parks, 205 W. Aztec Drive, Lewistown, MT 59457 USA; 4 U.S. Fish and Wildlife Service, Aquatic Animal Drug Approval Partnership Program, Bozeman Fish Technology Center, 4050 Bridger Canyon, Bozeman, MT 59715 USA; 5 Yellowstone Center for Resources, Yellowstone National Park, Wyoming, 82190 USA

**Keywords:** Environmental sciences, Conservation biology, Freshwater ecology

## Abstract

Despite unprecedented scientific productivity, Earth is undergoing a sixth mass extinction. The disconnect between scientific output and species conservation may be related to scientists studying the wrong species. Given fishes have a high extinction rate, we assessed the paradox between scientific productivity and science needed for conservation by comparing scientific output created for critically endangered fishes and game fishes. We searched 197,866 articles (1964–2018) in 112 journals for articles on 460 critically endangered fishes, 297 game fishes, and 35 fishes classified as critically endangered and game fish—our analysis included freshwater and marine species. Only 3% of the articles in the final database were on critically endangered fishes; 82% of critically endangered fishes had zero articles. The difference between the number of articles on game fishes and critically endangered fishes increased temporally with more articles on game fishes during the extinction crisis. Countries with 10 or more critically endangered fishes averaged only 17 articles from 1964 to 2018. Countries with the most critically endangered fishes are most in need of science. More scientific knowledge is needed on critically endangered fishes to meet the challenges of conserving fishes during the sixth mass extinction.

## Introduction

The human population increased by 133% from 1964 to 2018 and is expected to exceed 8 billion by 2023^1^. The increase in the number of humans on the planet and their activities have put an enormous strain on natural resources because humans destroy habitat, overexploit species, translocate invasive species, pollute, and disrupt the climate^2–5^. The dominating presence of human activity on Earth, particularly the influence of humans on the environment, has caused the adoption of a new geological era called the Anthropocene^6,7^, which some argue began during the Great Acceleration of the mid-twentieth century^8^. More specifically, others have coined the term Anthropocene Defaunation, which relates to the numerous extinctions caused by humans^9^. The mass extinction triggered by humans has caused scientists to believe the Earth is experiencing a sixth mass extinction^2,10–12^. Contemporary rates of extinction far exceed background rates^5,13^ and freshwater fishes had the highest extinction rate in the world among vertebrates in the twentieth century with an extinction rate in North America estimated at 877 times greater than the background extinction rate^14^.

Coincident with the Anthropocene and the sixth mass extinction, global scientific productivity, as defined by publication output, has increased exponentially^15–17^. The percent increase in the number of articles was 1,478% between 1964 and 2018 (Web of Science Core Collection searched year published 1964–2018) and the average doubling period of scientific literature was estimated at 15 years^17^. The development of scientific knowledge is multifaceted and is determined by complex interactions among social structures, societal problems, and knowledge previously gained. Growth in the scientific literature also does not necessarily equate to more conservation actions or better outcomes for species^18^; however, scientific knowledge is an important precursor to conservation^19,20^. Yet, there remains a mismatch between increasing extinction rates and the amount of scientific knowledge created for species conservation.

The increases in extinction rate and scientific knowledge created for species conservation is a unique paradox. The paradox could be a function of scientific knowledge not being used to reduce extinction rates, scientific knowledge not created on the species most in need, or a combination of both. Here we focus on whether the science knowledge created on fishes is for the species most in need of conservation. We used a bibliometric approach to measure the scientific output on fishes from 1964 (beginning of the IUCN Red List) through 2018 by comparing scientific output created for critically endangered fishes (defined by the International Union for Conservation of Nature [IUCN] as facing an extremely high risk of extinction in the wild in the immediate future), game fishes (defined by FishBase as a species targeted by anglers for recreational purposes), and fishes classified as critically endangered and game fish. We specifically addressed the following questions: is scientific knowledge created on fishes meeting the most pressing needs related to conservation of fishes in the context of mass extinction, and does the scientific knowledge created for critically endangered fishes match the geographical location for highest conservation need?

Scientific knowledge is the property of all humankind and there has been increased accountability of economic and social returns from public investments in science^16^. Evaluating the scientific enterprise can assist in addressing whether science is meeting societal needs. The science of science^16,17^ takes advantage of large data sets, such as the Web of Science, to evaluate the practice of science in search of widespread patterns such that science can effectively address problems that are important to society. Assessing the science on fisheries has been conducted by others^21–23^; however, our study is unique because we focus on scientific output from 1964 to 2018 as it relates to meeting the unprecedented challenges of conserving fishes during the sixth mass extinction.

## Results

**Figure 1:**
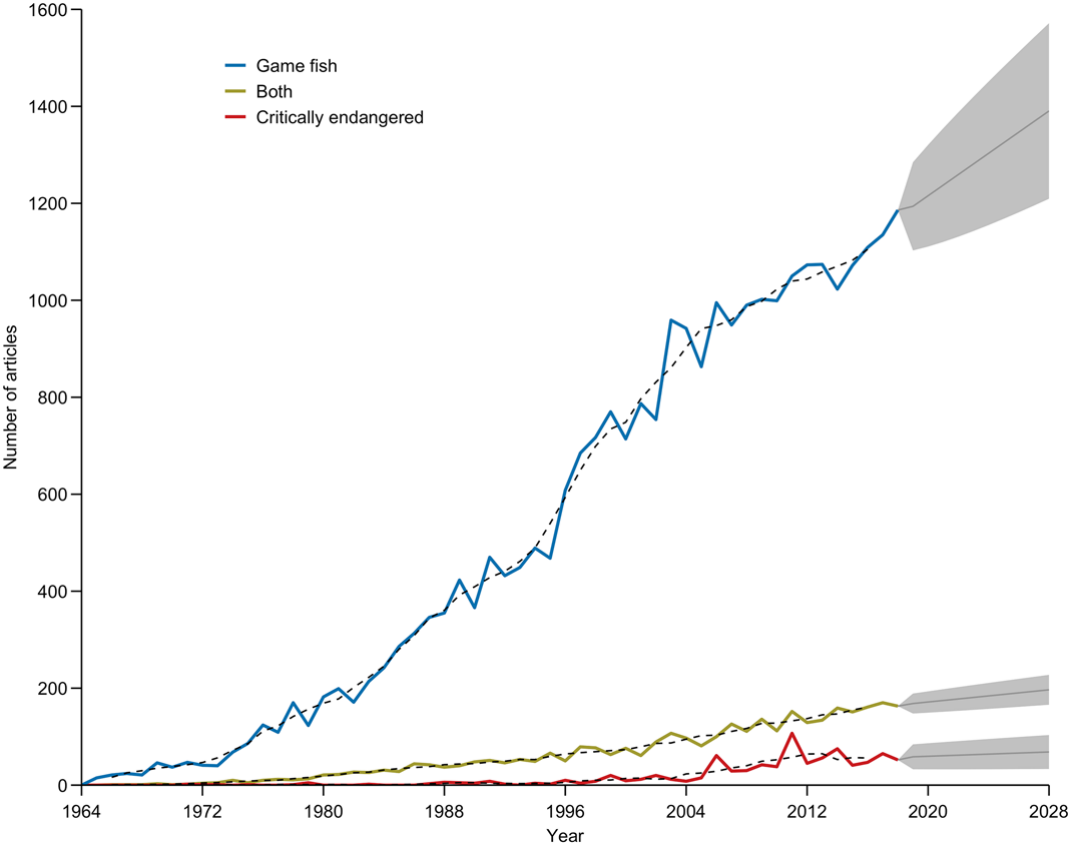
Number of articles published by year for fish species identified as game fish, both, and critically endangered fish. Game fish was defined by International Game Fish Association and listed by Donaldson et al. (2011) and critically endangered was defined by The International Union for Conservation of Nature (IUCN) Red List of Threatened Species. The category both includes species that were classified as game fish and critically endangered. Time series includes the 5-year moving average (dashed black lines) and 10-year forecasted number of articles (grey line) with 95% prediction intervals (grey ribbons). Figure was created using freely available R software (version 4.0.2 & URL: https://www.r-project.org/).

**Figure 2:**
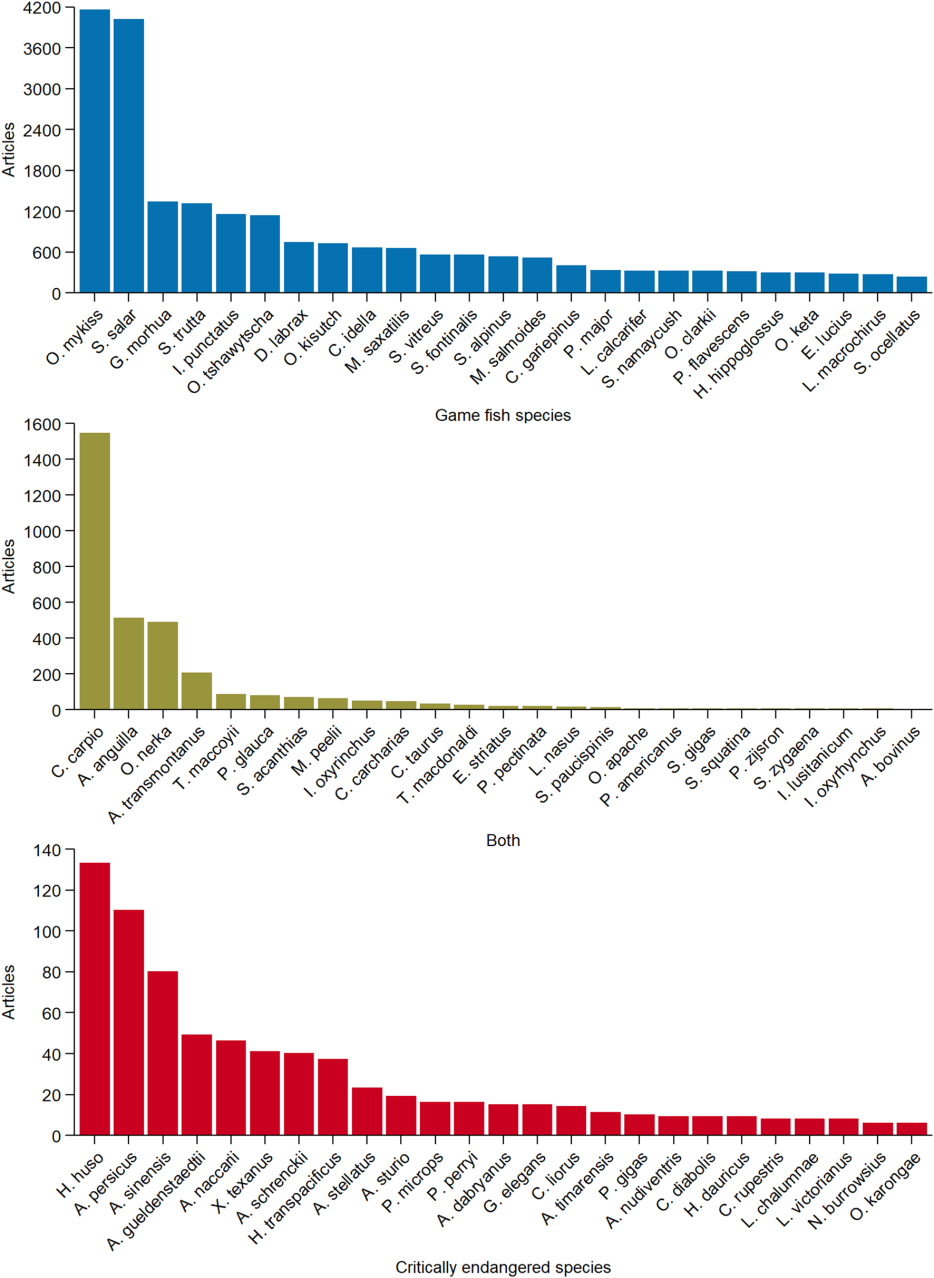
Number of articles from 1964 to 2018 by species for the top 25 game fish, both, and critically endangered fish. Game fish was defined by International Game Fish Association and listed by Donaldson et al. (2011) and critically endangered was defined by The International Union for Conservation of Nature (IUCN) Red List of Threatened Species. The category both includes species that were classified as game fish and critically endangered. See species list in supplemental material (table S1) for full scientific name. Figure was created using freely available R software (version 4.0.2 & URL: https://www.r-project.org/).

Of the 31,980 articles that we evaluated for the 792 species of interest (i.e., 460 critically endangered fishes, 297 game fishes, and 35 fishes classified as both), only 3% were on critically endangered fishes despite there being 163 more critically endangered fishes than game fishes in the fish-species database. The first articles on critically endangered fishes appeared in 1971 and the highest number of articles (N = 107) occurred in 2011 (Fig. 1). A change point in the number of articles on critically endangered species occurred in 1997 (1993–2003 CI). Prior to 1997, the average number of articles on critically endangered species per year was 1.8 (1.0–2.7 CI), subsequently, the average number of articles per year increased to 36.2 (24.5–47.8 CI). However, during the last decade the number of articles has remained level (slope = − 0.08, *P* = 0.97, df = 8) and the average number of articles forecasted in 2028 was 68.4 (33.4–103.4 PI; Fig. 1). Eighty-two percent of the critically endangered fishes had zero articles (table S1). Five percent of the critically endangered fishes constituted 85% of the articles, of those articles, 73% were on sturgeon species (order Acipenseriformes; Fig. 2). Six species (*Huso huso*, *Acipenser persicus*, *A. sinensis*, *A. gueldenstaedtii*, *A. naccarii*, and *Xyrauchen texanus*) composed 54% of all the articles on critically endangered fishes (Fig. 2).

Eighty-seven percent of the articles in the final database were on game fishes. The first articles on game fishes appeared in 1965, and the highest number of articles was 1,186 in 2018 (Fig. 1). A change point in the number of articles on game fishes occurred in 1979 (1976–1982 CI). Prior to 1979, the average number of articles on game fishes per year was 60.8 (35.0–86.5 CI), subsequently the average number of articles per year was 674.6 (567.0–782.3 CI). During the last decade the number of articles on game fish has increased (slope = 17.3, *P* = 0.0007, df = 8) and the average number of articles forecasted in 2028 was 1390.4 (1209.4–1571.5 PI; Fig. 1), which is 20.3 times higher than the number of articles forecasted for critically endangered fishes. Twenty percent of the game fishes had zero articles (table S1). Five percent of the game fishes composed 66% of all articles, with 65% of those articles on *Oncorhynchus mykiss*, *Salmo salar*, *Gadus morhua*, *Salmo trutta*, and *Ictalurus punctatus* (Fig. 2).

Ten percent of the articles were for species listed as both critically endangered and game fish. Fish species in both categories had slightly more articles than critically endangered fishes for all years and were noticeably lower than game fishes (Fig. 1). The first articles for fishes in both categories was in 1966, and the highest number of articles was 170 in 2017. A change point in the number of articles on fishes in both categories occurred in 1996 (1988–2002 CI). Prior to 1996, the average number of articles on fishes classified as both per year was 23.0 (15.9–30.2 CI), subsequently the average number of articles per year was 112.3 (96.5–128.2 CI). During the last decade the number of articles has increased (slope = 4.8, *P* = 0.007, df = 8) and the average number of articles forecasted in 2028 was 196.6 (165.8–227.5 PI; Fig. 1). Sixty-two percent of the articles for fish species listed as both critically endangered and game fish in both categories were on *Cyprinus carpio* and *Anguilla anguilla* (Fig. 2). The classification of *Cyprinus carpio* as critically endangered is geographic specific because it is based on a critically endangered subpopulation in the Danube River, otherwise the species is considered vulnerable by the IUCN.

**Figure 3:**
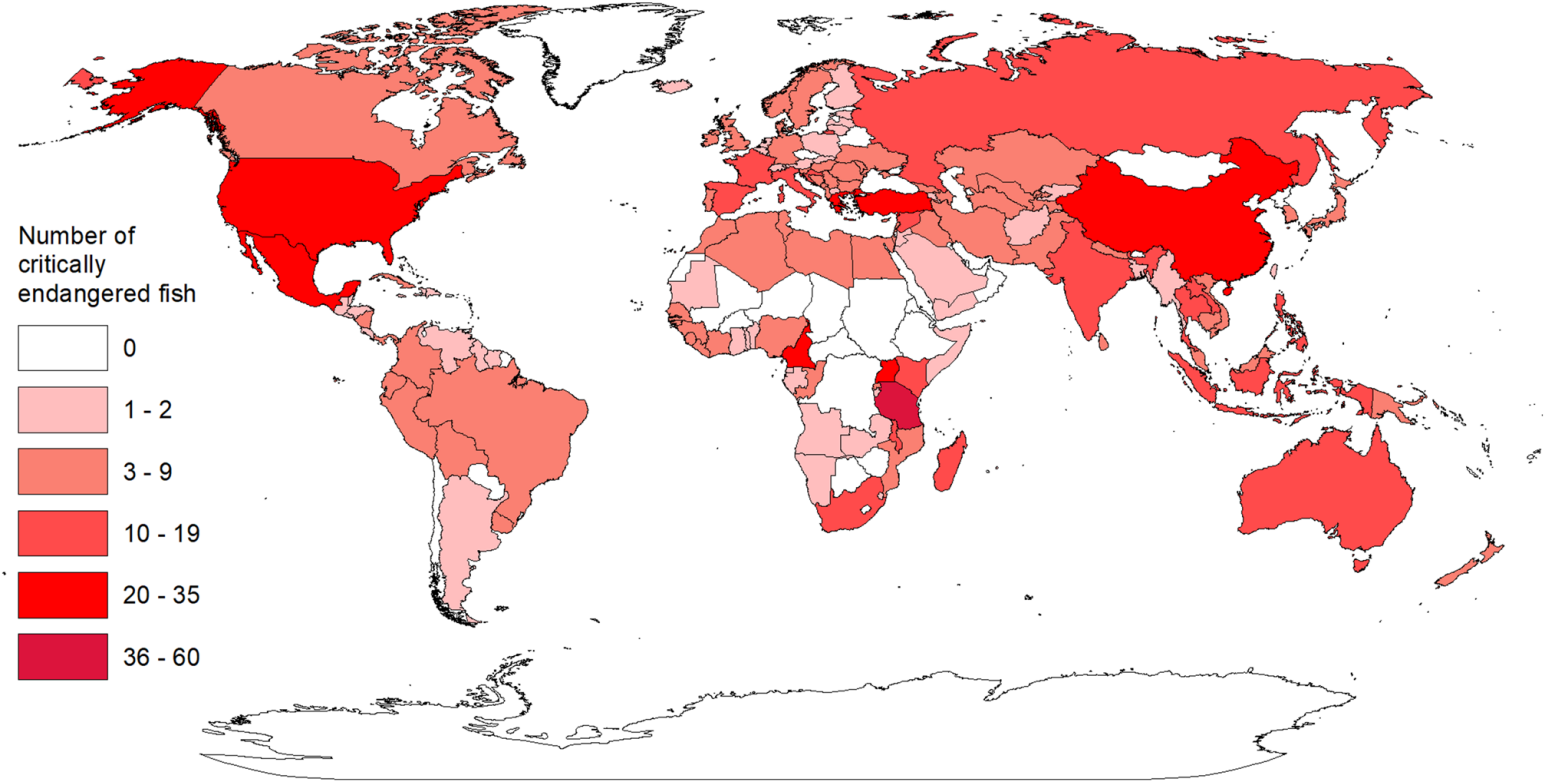
Number of critically endangered fish by country. Data were summarized from the International Union for Conservation of Nature (IUCN) Red List in 2018 for 460 critically endangered fishes. Figure was created using ArcMap (version 10.7.1 & URL: https://www.esri.com/en-us/home) and the outline of countries was acquired from the ArcGIS Hub (URL: https://hub.arcgis.com/datasets/a21fdb46d23e4ef896f31475217cbb08_1).

Tanzania had the highest number (N = 60) of critically endangered fishes, with Turkey, China, Mexico, Uganda, Greece, Cameroon, and the United States in the next category with 20–35 critically endangered fishes (Fig. 3). Sixteen countries were in the 10–19 category and 66 countries were in the 3–9 category. Twenty-three percent of the countries had zero critically endangered fishes (Fig. 3).

**Figure 4:**
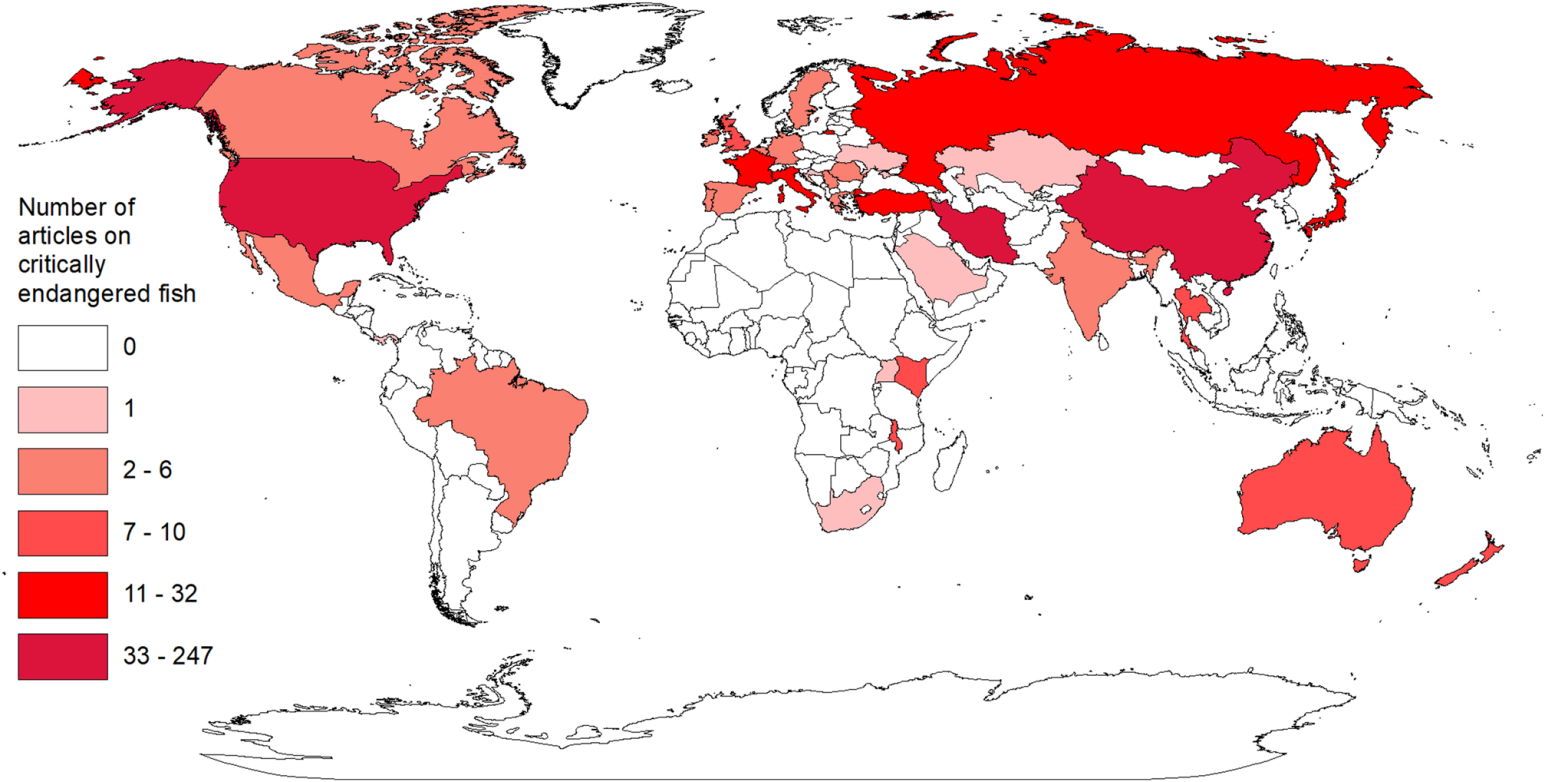
Number of articles on critically endangered fishes by country. Data are from 721 articles where any author on the article had a location that matched the geographical range of the critically endangered fish in the article title. Figure was created using ArcMap (version 10.7.1 & URL: https://www.esri.com/en-us/home) and the outline of countries was acquired from the ArcGIS Hub (URL: https://hub.arcgis.com/datasets/a21fdb46d23e4ef896f31475217cbb08_1).

Tanzania, Cameroon, Philippines, Madagascar, Laos, Syria, and Indonesia had 10 or more critically endangered fishes and zero articles on critically endangered fishes (Figs. 3 and 4). There were 114 countries (59%) that had at least one critically endangered fish and zero articles (Figs. 3 and 4). Iran (N = 247), United States (N = 137), and China (N = 110) had the highest number of articles on critically endangered fishes (Fig. 4), representing 69% of all articles on critically endangered fishes, despite the three countries only accounting for 14% of all critically endangered fishes. Furthermore, 89% of the articles on critically endangered fishes in Iran were on *Huso huso* (N = 114) and *Acipenser persicus* (N = 107), 57% of the articles on critically endangered fishes in the United States were on *Xyrauchen texanus* (N = 41) and *Hypomesus transpacificus* (N = 37), and 73% of the articles on critically endangered fishes in China were on *Acipenser sinensis* (N = 80). The top five countries in number of articles per number of critically endangered fishes within a country were Iran (35.2), United States (5.7), Japan (3.4), China (3.3), and Italy (2.2).

## Discussion

We contend that science on fishes is not meeting the most pressing needs related to conservation of fishes in the context of the sixth mass extinction. We demonstrated a vast mismatch between the growth in scientific output on fishes and the science for fishes most in need of conserving.

Much of the scientific output on fishes in the last five decades has been on game fishes. Furthermore, much of the research for critically endangered fishes and game fishes was skewed toward a few species and countries. We predicted that the sum of articles for game fishes would exceed critically endangered fishes but were surprised to find that the difference between the number of articles per year for game fishes and critically endangered fishes increased temporally and that the number of articles on critically endangered fishes plateaued during the last decade. We are at a crossroads in science needed for conservation of critically endangered fishes and argue for choosing the path of scientific productivity that matches the science needed for conservation practice.

Some disconcerting discoveries included that 82% of the critically endangered fishes in our database had zero articles and 23 species comprised 85% of the articles. Although a change point was detected and the rate of articles on critically endangered fishes has increased since 1997, the rate appears to have stabilized in the last decade and is woefully inadequate for science needed on all critically endangered fishes. We found that species classified as critically endangered and game fish had a slightly higher number of publications through time when compared to species classified solely as critically endangered, which indicates that having a recreational or commercial value may increase scientific output. Our results are conservative because we searched articles from the categories "Fisheries" and "Biodiversity and Conservation" in Web of Science and article titles for species names. Thus, the total number of articles for fishes within the categories used in this study were likely higher. In addition, our approach using InCites to select the journals for the Web of Science search resulted in mostly journals where the language was English, which may bias results^25,26^. Nevertheless, our results provide a reliable index of the fish species and countries that monopolize the scientific literature and illustrates the need for increased scientific output on critically endangered fishes.

We found that scientific knowledge created for critically endangered fishes does not match the geographical locations with the highest conservation need. That is, 59% of the countries with at least one critically endangered fish had zero articles. Moreover, three countries represented 69% of all the articles on critically endangered fishes. To analyze the disconnect between the countries with the most critically endangered fishes and countries that are conducting the most science on critically endangered fishes, we used a conservative approach and excluded the 15% of articles that did not have at least one author within the species range. We recognize that by excluding articles published by authors outside of a species range we are underrepresenting the number of articles on critically endangered fishes within a country; however, if we included those articles, it would over represent articles in the countries where the authors were located. The search for author location in relation to the species geographical range did give insight into how much research on critically endangered fishes is conducted by scientists outside a species range. Our analysis indicated that it is common for at least one author to occur in the geographical range of the critically endangered fish species. Thus, parachute science seems to be limited in the study of critically endangered fishes, which is not always the case in conservation science^27^. We continue to urge international collaborations because they are highly valuable for conservation efforts. However, researchers from outside countries need to involve scientists who are geographically matched to the area where the species in need of conservation occurs in order to ensure that science being conducted is relevant to the conservation planning processes^27–29^.

We recognize the need for science on game fishes, much of which is necessary to prevent overharvest of game-fish fisheries^24,30^. Our intention here is not to devalue the need for science on game fishes but to raise awareness for the need for science on critically endangered fishes relative to game fishes in the extinction crisis. Many of the top 25 game fish species in our analysis, for number of articles, are also considered a popular commercial species (e.g., *Oncorhynchus mykiss*, *Salmo salar*, *Gadus morhua*, *Salmo trutta*) and have a geographical range that includes North America. Thus, the model used to fund science for game fishes will likely not be transferable to critically endangered fishes at a global scale.

A fish-species approach was selected here to match the context of the sixth mass extinction and better understand the disparity in research productivity versus societal needs relative to conservation of critically endangered fishes. We argue that understanding the basic ecology of critically endangered species is vital to their conservation and single-species can be used as an umbrella species to conserve many co-occurring species^31,32^, which is valuable in increasing recognition for the freshwater biodiversity crisis^33,34^. Criteria for an umbrella species are rarity and sensitivity to human disturbance, which are identical to characteristics used for critically endangered species in this study^31^. Gaining support for critically endangered species will remain challenging because environmental issues often rank behind other problems faced by the public, such as the economy and health care, despite the growing public concern about the environment^29,35^. Funding is the limiting factor that prevents more research on critically endangered fishes and is most likely the cause of the dichotomy between science productivity for game fishes and critically endangered fishes observed in this study. Even in countries with high gross domestic product, such as the United States, funding for endangered species is inadequate^36^, and funding is often not related to priority needs for endangered species^37^. Moreover, charismatic species receive more funding and conservation attention without having recreational or commercial value^38^.

Much attention has been devoted to conservation practice and connecting science with policy^28,39,40^, but it has been argued that conservation practice is regularly not effective because conservation researchers often study issues not relevant to conservation practice or focus on easy conservation problems^39,41^. We contend the contemporary scientific knowledge created on fishes will not meet the scientific needs for conservation of fishes in the sixth mass extinction because the scientific output is not the science needed. This is corroborated by the United States National Research Council's statement that "…when science is gathered to inform environmental decisions, it is often not the right science"^41^. The results presented here underscore the immediate need for conducting the right science, which we define as science on critically endangered fishes. Conservation action based on rigorous science can prevent extinctions^19,20^. Moreover, addressing the geographic disconnect between researchers and areas with the highest conservation need for critically endangered fishes could result in more successful conservation actions^28,42^.

How do we increase funding for science to enhance our knowledge on critically endangered fishes? Numerous methods already exist that governmental agencies use to acquire funding for research on game fishes, such as excise taxes on fishing equipment, license sales, and Fisheries-based Ecotourism (FbA). A similar approach could be adopted for critically endangered fishes, or at a minimum, a portion of the proceeds from taxes, license sales, and FbA on game fishes could be allocated to critically endangered fishes. Tourism revenue from birdwatch has been highly successful in enhancing funding for conservation of critically endangered bird species^43^.

Decision analysis tools currently exist for allocating funds among endangered species that could possibly be adopted and adapted to balance funding between critically endangered fishes and game fishes^36,44^. Furthermore, given that monetary resources are limited to fund research on critically endangered fishes, one approach to prioritizing the allocation of funds would be to start where the largest disconnect between the number of critically endangered fishes and scientific knowledge exists^45^. If this approach were to be adopted, then funding for research on critically endangered fishes would be prioritized in Tanzania, Cameroon, Philippines, Madagascar, Laos, Syria, and Indonesia.

If history is used to inform the future, governmental agencies will not provide the resources required to ensure that science is focused on critically endangered fishes during the sixth mass extinction. We believe a global effort is necessary to diversify the portfolio of science on fishes and reverse the paradoxical knowledge gap. It will take partnerships between professional societies and non-governmental organizations to make a meaningful shift in the amount of science conducted on critically endangered fishes. Professional societies that facilitate the publication of science on fishes could partner with non-governmental organizations that focus on critically endangered species to assist in fund raising, prioritizing funding, and prioritize publishing science on critically endangered fishes. We posit four actions to promote and fund science on critically endangered fishes. First, the World Council of Fisheries Societies fosters coordination among scientists, professional societies, non-governmental organizations (e.g., IUCN, Fisheries Conservation Foundation [which currently partners with the American Fisheries Society], World Fish Migration Foundation, The Nature Conservancy, World Wildlife Fund), corporations (e.g., Google Sustainability Initiative), and citizen science organizations (e.g., Citizen Science Association) to promote and fund science on critically endangered fishes. Second, the World Council of Fisheries Societies and IUCN partner to provide an online marketplace where scientists can identify and apply for funding opportunities on critically endangered fishes (e.g., Save Our Species program, which established a program to save *Pseudobarbus burchelli* in the Cape Region of South Africa). Third, professional societies could work with The World Bank’s Environment, Natural Resources, and Blue Economy Global Practice Initiative to increase and prioritize science on critically endangered fishes in low- and middle-income countries. Fourth, professional societies could partner to reduce barriers to publishing by creating an no-fee, open-source journal called Endangered Fishes. Only partnerships at a global scale will ensure that we invest in the science that is needed for critically endangered fishes in the sixth mass extinction.

## Materials and methods

Names of freshwater and marine fish species were selected from two publicly available databases: the International Union for Conservation of Nature (IUCN) Red List of Threatened Species^46^ and the International Game Fish Association (IGFA)^47^ (fig. S1). Data for critically endangered fishes on the IUCN Red List in 2018 were collected using search filters in the advanced tab from the IUCN website—data gathered included genus, species, common name, and geographic range. Subspecies and species nova (newly identified species only described by a Genus; e.g., *Aplocheilichthys* sp. nov. Baringo) were excluded from the list of critically endangered species. Genus, species, and common name for game fishes recognized by the IGFA and listed by^24^ were collected from the IGFA website to create the game fishes database. Critically endangered fishes and game fishes databases were merged to create a fish-species database (fig. S1). Thirty-five species were classified as critically endangered and game fish (table S1) and were included in the category "Both" (table S1). Scientific names of fishes in the fish-species database were standardized to use the contemporary accepted scientific name provided by FishBase^48^. In addition, we included common names listed from all three data sources (IUCN, IGFA, and FishBase) and synonyms (which included historical binomial nomenclature) from FishBase (fig. S1). The final fish-species database included 460 critically endangered fishes, 297 game fishes, and 35 species classified as both, thus 792 species were used in the journal search (table S1).

InCites Journal Citation Reports (Clarivate Analytics) was used to select the journals (fig. S1). All journals in the categories "Fisheries" and "Biodiversity and Conservation," as defined by InCites Journal Citation Reports in 2018, were selected for the analysis, excluding the Journal of Shellfish Research because we focused on vertebrates (table S2). Web of Science Core Collection was used to select all articles from 1964 (first year of IUCN Red List) through 2018 from the 112 journals identified in the categories "Fisheries" and "Biodiversity and Conservation." We selected 1964 as the start date because the IUCN Red List of animals was first published in 1964. Metadata from 197,866 articles were downloaded and converted to a searchable format (i.e., CSV file) using R statistical software^49^ and the package bibliometrix^50^.

The titles of 197,866 articles were searched for critically endangered fishes and game fishes using the fish-species database (fig. S1). We only searched the titles of articles because searching abstracts and keywords within articles often included articles where a fish species was named but was not the focal species in the article, thus overrepresenting research conducted on a fish species. In addition, the inclusion of abstracts and keywords was not consistent among journals in the Web of Science and the inclusion of abstracts only occurred for more recent articles. Therefore, we used titles as an index to science productivity on critically endangered fishes and game fishes. We assumed the temporal use of species names in titles was similar between articles on critically endangered fishes and game fishes. Articles with two species names in the title were counted as one article for each species. There were cases where a common name of a fish was also the common name for another taxa. For example, an article on beluga whale *Delphinapterus leucas* would be selected because beluga for beluga sturgeon *Huso huso* would occur in the fish-species database. We removed all non-fish species from the database, which provided a final database of articles where critically endangered fishes, game fishes, and species classified as both were searched for in the title of the article (fig. S1).

The SiZer package^51^ in R was used to determine if a change point occurred in the number of articles through time for critically endangered fishes, game fishes, and both. If a change point was estimated, linear models were used to estimate the average rate of articles published per year before and after the change point. The 5-year moving average and 10-year forecast for the number of articles was estimated using the forecast package in R^52,53^. The auto.arima and checkresiduals functions were used to determine the best model. The ARIMA (0,1,1) with drift was the best model for all time series. All prediction intervals (PI) and confidence intervals (CI) are 95%.

The global distribution of critically endangered species was determined by searching each critically endangered fish species on the IUCN Red List website and recording the country(ies) listed in the geographic range—194 countries were included in the analysis. Global distribution of published articles on critically endangered fishes was conducted only for articles where the authors were within the geographical range of the species. Thus, we matched author location with the geographical range of the critically endangered fishes by using the list of countries where each critically endangered fish was located, and cross referenced every publication on the species list to determine if the country of at least one of the authors was on the list. If none of the authors were within the species geographical range, it was considered outside of the species range and filtered out for the global distribution of articles analysis. Eighty-five percent of the articles on critically endangered fishes had an author whose address was within the species range. This is a conservative approach to the global distribution of articles on critically endangered fishes, but better matches the magnitude of research conducted within the country of the critically endangered fishes and did not overrepresent countries where authors are working on critically endangered species outside their country.

Global distribution maps of critically endangered fishes and articles on critically endangered fishes were created using ArcMap 10.7.1 (ESRI 2019) and the outline of the countries was WGS 1984 from the ArcGIS Hub. We converted the projection to Eckert III. Breaks for symbology were determined by using Jenks natural breaks classification method with five categories. The use of Jenks natural breaks allows for representation of categories based on gaps identified from frequency histograms to maximize the variance among categories and minimize the variance within categories^54,55^. Additionally, a sixth category was added to both maps to delineate locations with zero critically endangered fishes and articles.

## Supplementary Information

Supplementary Information

## Data Availability

All data needed to evaluate the conclusions in the paper are present in the paper or Supplementary Materials. Electronic versions of the data may be requested from the authors.
