# Supplementary material for: A paradoxical knowledge gap in science for critically endangered fishes and game fishes during the sixth mass extinction
Source: Sci Rep. 2021 Apr 19;11:8447. doi: 10.1038/s41598-021-87871-y (PMC8055981; doi:10.1038/s41598-021-87871-y)
Supplement: Supplementary file 1 — Supplementary Information [file 41598_2021_87871_MOESM1_ESM.docx]

A paradoxical knowledge gap in science for critically endangered fishes and game fishes during the sixth mass extinction

Authors:

Christopher S. Guy^1*^, Tanner L. Cox^2^, Jacob R. Williams^2†^, Colter D. Brown^2^, Robert W. Eckelbecker^2^, Hayley C. Glassic^2^, Madeline C. Lewis^2^, Paige A. C. Maskill^2‡^, Lauren M. McGarvey^2§^, and Michael J. Siemiantkowski^2^

^1^U.S. Geological Survey, Montana Cooperative Fishery Research Unit, Department of Ecology, Montana State University, PO Box 173460, Bozeman, Montana 59717 USA. cguy@montana.edu

^2^Montana Cooperative Fishery Research Unit, Department of Ecology, Montana State University, PO Box 173460, Bozeman, Montana 59717 USA.

*cguy@montana.edu

^†^Montana Fish, Wildlife and Parks, 205 W. Aztec Drive, Lewistown, Montana 59457

^‡^U.S. Fish and Wildlife Service, Aquatic Animal Drug Approval Partnership Program, Bozeman Fish Technology Center, 4050 Bridger Canyon, Bozeman, Montana 59715

^§^Yellowstone Center for Resources, Yellowstone National Park, Wyoming 82190

## Supplemental Tables

Table S1. Scientific name, status (critically endangered was identified from the International Union for Conservation of Nature (IUCN) Red List in 2018, game fish was identified from the International Game Fish Association (IGFA) list of recognized game fishes of 2018 and Donaldson et al. (2011), and both when species were classified as critically endangered and game fish), and number of published articles (see methods for how published article data were collected) for 460 critically endangered species, 297 game fish species, and 35 species classified as both.

| Species | | Status | | Articles | |
| --- | --- | --- | --- | --- | --- |
|  | |  |  | | |
| *Aaptosyax grypus* | Critically endangered | | 0 |  |  |
| *Acanthobrama centisquama* | Critically endangered | | 0 |  |  |
| *Acanthobrama hadiyahensis* | Critically endangered | | 1 |  |  |
| *Acanthobrama tricolor* | Critically endangered | | 0 |  |  |
| *Acanthocybium solandri* | Game fish | | 10 |  |  |
| *Acheilognathus elongatus* | Critically endangered | | 0 |  |  |
| *Acipenser dabryanus* | Critically endangered | | 15 |  |  |
| *Acipenser gueldenstaedtii* | Critically endangered | | 49 |  |  |
| *Acipenser mikadoi* | Critically endangered | | 3 |  |  |
| *Acipenser naccarii* | Critically endangered | | 46 |  |  |
| *Acipenser nudiventris* | Critically endangered | | 9 |  |  |
| *Acipenser persicus* | Critically endangered | | 110 |  |  |
| *Acipenser schrenckii* | Critically endangered | | 40 |  |  |
| *Acipenser sinensis* | Critically endangered | | 80 |  |  |
| *Acipenser stellatus* | Critically endangered | | 23 |  |  |
| *Acipenser sturio* | Critically endangered | | 19 |  |  |
| *Acipenser transmontanus* | Both | | 206 |  |  |
| *Acroteriobatus variegatus* | Critically endangered | | 0 |  |  |
| *Adrianichthys kruyti* | Critically endangered | | 0 |  |  |
| *Adrianichthys poptae* | Critically endangered | | 0 |  |  |
| *Aetomylaeus bovinus* | Both | | 1 |  |  |
| *Akihito futuna* | Critically endangered | | 0 |  |  |
| *Albula argentea* | Game fish | | 0 |  |  |
| *Albula glossodonta* | Game fish | | 0 |  |  |
| *Albula nemoptera* | Game fish | | 0 |  |  |
| *Albula vulpes* | Game fish | | 26 |  |  |
| *Alburnus macedonicus* | Critically endangered | | 0 |  |  |
| *Alburnus mandrensis* | Critically endangered | | 0 |  |  |
| *Alburnus nasreddini* | Critically endangered | | 1 |  |  |
| *Alburnus timarensis* | Critically endangered | | 11 |  |  |
| *Alburnus vistonicus* | Critically endangered | | 0 |  |  |
| *Alectis ciliaris* | Game fish | | 0 |  |  |
| *Allotoca diazi* | Critically endangered | | 0 |  |  |
| *Allotoca maculata* | Critically endangered | | 0 |  |  |
| *Alopias pelagicus* | Game fish | | 4 |  |  |
| *Alopias superciliosus* | Game fish | | 12 |  |  |
| *Alopias vulpinus* | Game fish | | 17 |  |  |
| *Alosa killarnensis* | Critically endangered | | 0 |  |  |
| *Alosa sapidissima* | Game fish | | 116 |  |  |
| *Alosa vistonica* | Critically endangered | | 0 |  |  |
| *Ambassis ambassis* | Game fish | | 0 |  |  |
| *Ambloplites rupestris* | Game fish | | 19 |  |  |
| *Ameiurus catus* | Game fish | | 8 |  |  |
| *Ameiurus melas* | Game fish | | 31 |  |  |
| *Ameiurus natalis* | Game fish | | 2 |  |  |
| *Ameiurus nebulosus* | Game fish | | 36 |  |  |
| *Amia calva* | Game fish | | 8 |  |  |
| *Amphilophus zaliosus* | Critically endangered | | 0 |  |  |
| *Anabarilius andersoni* | Critically endangered | | 0 |  |  |
| *Anabarilius qiluensis* | Critically endangered | | 0 |  |  |
| *Anabarilius yangzonensis* | Critically endangered | | 0 |  |  |
| *Anablepsoides speciosus* | Critically endangered | | 0 |  |  |
| *Anguilla anguilla* | Both | | 512 |  |  |
| *Aphanius almiriensis* | Critically endangered | | 0 |  |  |
| *Aphanius danfordii* | Critically endangered | | 2 |  |  |
| *Aphanius saourensis* | Critically endangered | | 0 |  |  |
| *Aphanius sirhani* | Critically endangered | | 0 |  |  |
| *Aphanius transgrediens* | Critically endangered | | 0 |  |  |
| *Aphyolebias claudiae* | Critically endangered | | 0 |  |  |
| *Aplodinotus grunniens* | Game fish | | 17 |  |  |
| *Apogon imberbis* | Game fish | | 5 |  |  |
| *Aposturisoma myriodon* | Critically endangered | | 0 |  |  |
| *Argyrosomus regius* | Game fish | | 87 |  |  |
| *Arius festinus* | Critically endangered | | 0 |  |  |
| *Arius uncinatus* | Critically endangered | | 0 |  |  |
| *Arripis trutta* | Game fish | | 10 |  |  |
| *Astroblepus formosus* | Critically endangered | | 0 |  |  |
| *Astroblepus ubidiai* | Critically endangered | | 2 |  |  |
| *Astronotus ocellatus* | Game fish | | 13 |  |  |
| *Atherinella jiloaensis* | Critically endangered | | 0 |  |  |
| *Atractoscion nobilis* | Game fish | | 48 |  |  |
| *Atractosteus spatula* | Game fish | | 29 |  |  |
| *Aulonocara baenschi* | Critically endangered | | 0 |  |  |
| *Aulonocara kandeensis* | Critically endangered | | 0 |  |  |
| *Aulonocara maylandi* | Critically endangered | | 0 |  |  |
| *Austrolebias cinereus* | Critically endangered | | 0 |  |  |
| *Azurina eupalama* | Critically endangered | | 0 |  |  |
| *Bagrus meridionalis* | Critically endangered | | 1 |  |  |
| *Bahaba taipingensis* | Critically endangered | | 0 |  |  |
| *Balantiocheilos ambusticauda* | Critically endangered | | 0 |  |  |
| *Bangana decora* | Critically endangered | | 0 |  |  |
| *Barbodes amarus* | Critically endangered | | 0 |  |  |
| *Barbodes baoulan* | Critically endangered | | 0 |  |  |
| *Barbodes clemensi* | Critically endangered | | 0 |  |  |
| *Barbodes disa* | Critically endangered | | 0 |  |  |
| *Barbodes flavifuscus* | Critically endangered | | 0 |  |  |
| *Barbodes herrei* | Critically endangered | | 0 |  |  |
| *Barbodes katolo* | Critically endangered | | 0 |  |  |
| *Barbodes lanaoensis* | Critically endangered | | 0 |  |  |
| *Barbodes manalak* | Critically endangered | | 0 |  |  |
| *Barbodes pachycheilus* | Critically endangered | | 0 |  |  |
| *Barbodes palata* | Critically endangered | | 0 |  |  |
| *Barbodes resinus* | Critically endangered | | 0 |  |  |
| *Barbodes tras* | Critically endangered | | 0 |  |  |
| *Barbodes truncatulus* | Critically endangered | | 0 |  |  |
| *Barbodes wynaadensis* | Critically endangered | | 0 |  |  |
| *Barbus euboicus* | Critically endangered | | 0 |  |  |
| *Bedotia tricolor* | Critically endangered | | 0 |  |  |
| *Betta miniopinna* | Critically endangered | | 0 |  |  |
| *Betta persephone* | Critically endangered | | 0 |  |  |
| *Betta simplex* | Critically endangered | | 0 |  |  |
| *Betta spilotogena* | Critically endangered | | 0 |  |  |
| *Brachionichthys hirsutus* | Critically endangered | | 0 |  |  |
| *Brienomyrus longianalis* | Critically endangered | | 1 |  |  |
| *Caecocypris basimi* | Critically endangered | | 0 |  |  |
| *Callionymus bairdi* | Critically endangered | | 0 |  |  |
| *Capoeta pestai* | Critically endangered | | 0 |  |  |
| *Caranx caninus* | Game fish | | 0 |  |  |
| *Caranx hippos* | Game fish | | 3 |  |  |
| *Caranx ignobilis* | Game fish | | 3 |  |  |
| *Caranx latus* | Game fish | | 1 |  |  |
| *Caranx melampygus* | Game fish | | 7 |  |  |
| *Caranx sexfasciatus* | Game fish | | 1 |  |  |
| *Carcharhinus acronotus* | Game fish | | 5 |  |  |
| *Carcharhinus albimarginatus* | Game fish | | 1 |  |  |
| *Carcharhinus altimus* | Game fish | | 1 |  |  |
| *Carcharhinus amblyrhynchoides* | Game fish | | 0 |  |  |
| *Carcharhinus amblyrhynchos* | Game fish | | 2 |  |  |
| *Carcharhinus amboinensis* | Game fish | | 2 |  |  |
| *Carcharhinus borneensis* | Game fish | | 0 |  |  |
| *Carcharhinus brachyurus* | Game fish | | 4 |  |  |
| *Carcharhinus brevipinna* | Game fish | | 1 |  |  |
| *Carcharhinus cautus* | Game fish | | 1 |  |  |
| *Carcharhinus dussumieri* | Game fish | | 2 |  |  |
| *Carcharhinus falciformis* | Game fish | | 15 |  |  |
| *Carcharhinus fitzroyensis* | Game fish | | 0 |  |  |
| *Carcharhinus galapagensis* | Game fish | | 2 |  |  |
| *Carcharhinus hemiodon* | Both | | 0 |  |  |
| *Carcharhinus isodon* | Game fish | | 4 |  |  |
| *Carcharhinus leiodon* | Game fish | | 2 |  |  |
| *Carcharhinus leucas* | Game fish | | 27 |  |  |
| *Carcharhinus limbatus* | Game fish | | 23 |  |  |
| *Carcharhinus longimanus* | Game fish | | 7 |  |  |
| *Carcharhinus macloti* | Game fish | | 0 |  |  |
| *Carcharhinus melanopterus* | Game fish | | 11 |  |  |
| *Carcharhinus obscurus* | Game fish | | 12 |  |  |
| *Carcharhinus perezii* | Game fish | | 2 |  |  |
| *Carcharhinus plumbeus* | Game fish | | 39 |  |  |
| *Carcharhinus porosus* | Game fish | | 3 |  |  |
| *Carcharhinus sealei* | Game fish | | 0 |  |  |
| *Carcharhinus signatus* | Game fish | | 2 |  |  |
| *Carcharhinus sorrah* | Game fish | | 2 |  |  |
| *Carcharhinus tilstoni* | Game fish | | 6 |  |  |
| *Carcharias taurus* | Both | | 30 |  |  |
| *Carcharodon carcharias* | Both | | 46 |  |  |
| *Caspiomyzon hellenicus* | Critically endangered | | 0 |  |  |
| *Catlocarpio siamensis* | Critically endangered | | 0 |  |  |
| *Centrophorus granulosus* | Critically endangered | | 4 |  |  |
| *Centropomus armatus* | Game fish | | 1 |  |  |
| *Centropomus ensiferus* | Game fish | | 1 |  |  |
| *Centropomus medius* | Game fish | | 2 |  |  |
| *Centropomus mexicanus* | Game fish | | 0 |  |  |
| *Centropomus nigrescens* | Game fish | | 1 |  |  |
| *Centropomus parallelus* | Game fish | | 34 |  |  |
| *Centropomus pectinatus* | Game fish | | 1 |  |  |
| *Centropomus poeyi* | Game fish | | 0 |  |  |
| *Centropomus robalito* | Game fish | | 0 |  |  |
| *Centropomus undecimalis* | Game fish | | 49 |  |  |
| *Centropomus unionensis* | Game fish | | 0 |  |  |
| *Centropomus viridis* | Game fish | | 0 |  |  |
| *Centropristis striata* | Game fish | | 64 |  |  |
| *Ceratoglanis pachynema* | Critically endangered | | 0 |  |  |
| *Chaetodipterus faber* | Game fish | | 5 |  |  |
| *Channa amphibeus* | Game fish | | 0 |  |  |
| *Channa argus* | Game fish | | 57 |  |  |
| *Channa asiatica* | Game fish | | 0 |  |  |
| *Channa aurantimaculata* | Game fish | | 1 |  |  |
| *Channa bankanensis* | Game fish | | 0 |  |  |
| *Channa baramensis* | Game fish | | 0 |  |  |
| *Channa barca* | Game fish | | 0 |  |  |
| *Channa bleheri* | Game fish | | 1 |  |  |
| *Channa burmanica* | Game fish | | 0 |  |  |
| *Channa cyanospilos* | Game fish | | 0 |  |  |
| *Channa diplogramma* | Game fish | | 0 |  |  |
| *Channa gachua* | Game fish | | 3 |  |  |
| *Channa harcourtbutleri* | Game fish | | 0 |  |  |
| *Channa lucius* | Game fish | | 0 |  |  |
| *Channa maculata* | Game fish | | 14 |  |  |
| *Channa marulioides* | Game fish | | 0 |  |  |
| *Channa marulius* | Game fish | | 5 |  |  |
| *Channa melanoptera* | Game fish | | 0 |  |  |
| *Channa melasoma* | Game fish | | 0 |  |  |
| *Channa micropeltes* | Game fish | | 3 |  |  |
| *Channa nox* | Game fish | | 0 |  |  |
| *Channa orientalis* | Game fish | | 0 |  |  |
| *Channa panaw* | Game fish | | 0 |  |  |
| *Channa pleurophthalma* | Game fish | | 0 |  |  |
| *Channa punctata* | Game fish | | 64 |  |  |
| *Channa stewartii* | Game fish | | 2 |  |  |
| *Channa striata* | Game fish | | 78 |  |  |
| *Chasmistes liorus* | Critically endangered | | 14 |  |  |
| *Cheilodipterus arabicus* | Game fish | | 0 |  |  |
| *Cheilodipterus macrodon* | Game fish | | 0 |  |  |
| *Chilatherina sentaniensis* | Critically endangered | | 0 |  |  |
| *Chiloglanis bifurcus* | Critically endangered | | 0 |  |  |
| *Chiloglanis polyodon* | Critically endangered | | 0 |  |  |
| *Chiloglanis ruziziensis* | Critically endangered | | 0 |  |  |
| *Chindongo saulosi* | Critically endangered | | 0 |  |  |
| *Chlamydogobius micropterus* | Critically endangered | | 0 |  |  |
| *Chlamydogobius squamigenus* | Critically endangered | | 0 |  |  |
| *Chrysoblephus cristiceps* | Both | | 0 |  |  |
| *Cichla intermedia* | Game fish | | 0 |  |  |
| *Cichla ocellaris* | Game fish | | 9 |  |  |
| *Cichla temensis* | Game fish | | 6 |  |  |
| *Clarias cavernicola* | Critically endangered | | 0 |  |  |
| *Clarias gariepinus* | Game fish | | 397 |  |  |
| *Clarias maclareni* | Critically endangered | | 0 |  |  |
| *Clupeonella abrau* | Critically endangered | | 0 |  |  |
| *Cobitis illyrica* | Critically endangered | | 0 |  |  |
| *Cobitis jadovaensis* | Critically endangered | | 0 |  |  |
| *Cobitis kellei* | Critically endangered | | 0 |  |  |
| *Cobitis puncticulata* | Critically endangered | | 0 |  |  |
| *Cobitis splendens* | Critically endangered | | 0 |  |  |
| *Cobitis stephanidisi* | Critically endangered | | 0 |  |  |
| *Cobitis taurica* | Critically endangered | | 0 |  |  |
| *Colossoma macropomum* | Game fish | | 101 |  |  |
| *Conger conger* | Game fish | | 7 |  |  |
| *Copadichromis nkatae* | Critically endangered | | 0 |  |  |
| *Coptodon coffea* | Critically endangered | | 0 |  |  |
| *Coregonus bavaricus* | Critically endangered | | 0 |  |  |
| *Coregonus clupeaformis* | Game fish | | 98 |  |  |
| *Coregonus hoferi* | Critically endangered | | 0 |  |  |
| *Coregonus huntsmani* | Critically endangered | | 2 |  |  |
| *Coregonus pennantii* | Critically endangered | | 1 |  |  |
| *Coregonus reighardi* | Critically endangered | | 0 |  |  |
| *Coregonus trybomi* | Critically endangered | | 0 |  |  |
| *Corematodus shiranus* | Critically endangered | | 0 |  |  |
| *Coryphaena hippurus* | Game fish | | 59 |  |  |
| *Coryphaenoides rupestris* | Critically endangered | | 8 |  |  |
| *Cottus paulus* | Critically endangered | | 3 |  |  |
| *Cottus rondeleti* | Critically endangered | | 0 |  |  |
| *Crystallaria cincotta* | Critically endangered | | 4 |  |  |
| *Ctenopharyngodon idella* | Game fish | | 661 |  |  |
| *Ctenopoma ocellatum* | Critically endangered | | 0 |  |  |
| *Cynoscion nebulosus* | Game fish | | 54 |  |  |
| *Cynoscion regalis* | Game fish | | 42 |  |  |
| *Cyprinella alvarezdelvillari* | Critically endangered | | 0 |  |  |
| *Cyprinella bocagrande* | Critically endangered | | 0 |  |  |
| *Cyprinodon diabolis* | Critically endangered | | 9 |  |  |
| *Cyprinodon labiosus* | Critically endangered | | 0 |  |  |
| *Cyprinodon meeki* | Critically endangered | | 0 |  |  |
| *Cyprinodon pachycephalus* | Critically endangered | | 0 |  |  |
| *Cyprinodon verecundus* | Critically endangered | | 0 |  |  |
| *Cyprinodon veronicae* | Critically endangered | | 0 |  |  |
| *Cyprinus barbatus* | Critically endangered | | 0 |  |  |
| *Cyprinus carpio* | Both | | 1544 |  |  |
| *Cyprinus fuxianensis* | Critically endangered | | 0 |  |  |
| *Cyprinus ilishaestomus* | Critically endangered | | 0 |  |  |
| *Cyprinus micristius* | Critically endangered | | 0 |  |  |
| *Cyprinus qionghaiensis* | Critically endangered | | 0 |  |  |
| *Cyprinus yunnanensis* | Critically endangered | | 0 |  |  |
| *Datnioides pulcher* | Critically endangered | | 0 |  |  |
| *Delminichthys jadovensis* | Critically endangered | | 0 |  |  |
| *Delminichthys krbavensis* | Critically endangered | | 0 |  |  |
| *Dentex dentex* | Game fish | | 103 |  |  |
| *Dicentrarchus labrax* | Game fish | | 735 |  |  |
| *Dipturus batis* | Both | | 0 |  |  |
| *Dipturus confusus* | Critically endangered | | 0 |  |  |
| *Elagatis bipinnulata* | Game fish | | 0 |  |  |
| *Elassoma alabamae* | Critically endangered | | 0 |  |  |
| *Electrolux addisoni* | Critically endangered | | 0 |  |  |
| *Empetrichthys latos* | Critically endangered | | 1 |  |  |
| *Encheloclarias curtisoma* | Critically endangered | | 0 |  |  |
| *Encheloclarias kelioides* | Critically endangered | | 0 |  |  |
| *Enteromius boboi* | Critically endangered | | 0 |  |  |
| *Enteromius carcharhinoides* | Critically endangered | | 0 |  |  |
| *Enteromius melanotaenia* | Critically endangered | | 0 |  |  |
| *Enteromius treurensis* | Critically endangered | | 0 |  |  |
| *Epalzeorhynchos bicolor* | Critically endangered | | 1 |  |  |
| *Epinephelus itajara* | Game fish | | 16 |  |  |
| *Epinephelus morio* | Game fish | | 34 |  |  |
| *Epinephelus striatus* | Both | | 17 |  |  |
| *Epiplatys coccinatus* | Critically endangered | | 0 |  |  |
| *Epiplatys ruhkopfi* | Critically endangered | | 0 |  |  |
| *Eptatretus octatrema* | Critically endangered | | 0 |  |  |
| *Esox lucius* | Game fish | | 277 |  |  |
| *Esox masquinongy* | Game fish | | 90 |  |  |
| *Esox niger* | Game fish | | 1 |  |  |
| *Etheostoma chermocki* | Critically endangered | | 0 |  |  |
| *Etheostoma marmorpinnum* | Critically endangered | | 0 |  |  |
| *Etheostoma percnurum* | Critically endangered | | 0 |  |  |
| *Eusphyra blochii* | Game fish | | 0 |  |  |
| *Euthynnus affinis* | Game fish | | 14 |  |  |
| *Euthynnus alletteratus* | Game fish | | 8 |  |  |
| *Euthynnus lineatus* | Game fish | | 1 |  |  |
| *Fundulopanchax powelli* | Critically endangered | | 0 |  |  |
| *Gadus macrocephalus* | Game fish | | 79 |  |  |
| *Gadus morhua* | Game fish | | 1332 |  |  |
| *Galaxias anomalus* | Critically endangered | | 1 |  |  |
| *Galaxias cobitinis* | Critically endangered | | 0 |  |  |
| *Galaxias eldoni* | Critically endangered | | 0 |  |  |
| *Galaxias fontanus* | Critically endangered | | 0 |  |  |
| *Galaxias fuscus* | Critically endangered | | 5 |  |  |
| *Galaxias gollumoides* | Critically endangered | | 0 |  |  |
| *Galaxias johnstoni* | Critically endangered | | 0 |  |  |
| *Galaxias macronasus* | Critically endangered | | 0 |  |  |
| *Galaxias pedderensis* | Critically endangered | | 1 |  |  |
| *Galaxias prognathus* | Critically endangered | | 2 |  |  |
| *Galaxias pullus* | Critically endangered | | 0 |  |  |
| *Galeocerdo cuvier* | Game fish | | 30 |  |  |
| *Galeorhinus galeus* | Game fish | | 21 |  |  |
| *Gambusia eurystoma* | Critically endangered | | 1 |  |  |
| *Garra festai* | Critically endangered | | 0 |  |  |
| *Garra ornata* | Critically endangered | | 0 |  |  |
| *Garra widdowsoni* | Critically endangered | | 0 |  |  |
| *Gila elegans* | Critically endangered | | 15 |  |  |
| *Gila modesta* | Critically endangered | | 0 |  |  |
| *Girardinichthys viviparus* | Critically endangered | | 1 |  |  |
| *Glossolepis wanamensis* | Critically endangered | | 0 |  |  |
| *Glyphis gangeticus* | Both | | 1 |  |  |
| *Glyphis garricki* | Critically endangered | | 1 |  |  |
| *Glyphis glyphis* | Game fish | | 1 |  |  |
| *Glyphis siamensis* | Both | | 0 |  |  |
| *Glyptothorax kashmirensis* | Critically endangered | | 1 |  |  |
| *Glyptothorax kudremukhensis* | Critically endangered | | 0 |  |  |
| *Gobio delyamurei* | Critically endangered | | 0 |  |  |
| *Gobio gymnostethus* | Critically endangered | | 0 |  |  |
| *Gobio hettitorum* | Critically endangered | | 0 |  |  |
| *Gobio insuyanus* | Critically endangered | | 0 |  |  |
| *Gobulus birdsongi* | Critically endangered | | 0 |  |  |
| *Gymnocephalus ambriaelacus* | Critically endangered | | 0 |  |  |
| *Gymnosarda unicolor* | Game fish | | 2 |  |  |
| *Gymnura altavela* | Both | | 0 |  |  |
| *Hampala lopezi* | Critically endangered | | 0 |  |  |
| *Haploblepharus kistnasamyi* | Critically endangered | | 0 |  |  |
| *Haplochromis aelocephalus* | Critically endangered | | 0 |  |  |
| *Haplochromis annectidens* | Critically endangered | | 0 |  |  |
| *Haplochromis antleter* | Critically endangered | | 0 |  |  |
| *Haplochromis apogonoides* | Critically endangered | | 0 |  |  |
| *Haplochromis argenteus* | Critically endangered | | 0 |  |  |
| *Haplochromis barbarae* | Critically endangered | | 0 |  |  |
| *Haplochromis bareli* | Critically endangered | | 0 |  |  |
| *Haplochromis beadlei* | Critically endangered | | 0 |  |  |
| *Haplochromis brownae* | Critically endangered | | 0 |  |  |
| *Haplochromis cassius* | Critically endangered | | 0 |  |  |
| *Haplochromis cinctus* | Critically endangered | | 0 |  |  |
| *Haplochromis cnester* | Critically endangered | | 0 |  |  |
| *Haplochromis coprologus* | Critically endangered | | 0 |  |  |
| *Haplochromis crassilabris* | Critically endangered | | 0 |  |  |
| *Haplochromis crocopeplus* | Critically endangered | | 0 |  |  |
| *Haplochromis dentex* | Critically endangered | | 0 |  |  |
| *Haplochromis dichrourus* | Critically endangered | | 0 |  |  |
| *Haplochromis flavipinnis* | Critically endangered | | 0 |  |  |
| *Haplochromis granti* | Critically endangered | | 0 |  |  |
| *Haplochromis guiarti* | Critically endangered | | 0 |  |  |
| *Haplochromis heusinkveldi* | Critically endangered | | 0 |  |  |
| *Haplochromis hiatus* | Critically endangered | | 0 |  |  |
| *Haplochromis iris* | Critically endangered | | 0 |  |  |
| *Haplochromis ishmaeli* | Critically endangered | | 0 |  |  |
| *Haplochromis katunzii* | Critically endangered | | 0 |  |  |
| *Haplochromis latifasciatus* | Critically endangered | | 0 |  |  |
| *Haplochromis longirostris* | Critically endangered | | 0 |  |  |
| *Haplochromis macrognathus* | Critically endangered | | 0 |  |  |
| *Haplochromis martini* | Critically endangered | | 0 |  |  |
| *Haplochromis michaeli* | Critically endangered | | 0 |  |  |
| *Haplochromis microdon* | Critically endangered | | 0 |  |  |
| *Haplochromis mylergates* | Critically endangered | | 0 |  |  |
| *Haplochromis nanoserranus* | Critically endangered | | 0 |  |  |
| *Haplochromis obesus* | Critically endangered | | 0 |  |  |
| *Haplochromis pancitrinus* | Critically endangered | | 0 |  |  |
| *Haplochromis parvidens* | Critically endangered | | 0 |  |  |
| *Haplochromis percoides* | Critically endangered | | 0 |  |  |
| *Haplochromis perrieri* | Critically endangered | | 0 |  |  |
| *Haplochromis plutonius* | Critically endangered | | 0 |  |  |
| *Haplochromis ptistes* | Critically endangered | | 0 |  |  |
| *Haplochromis pyrrhopteryx* | Critically endangered | | 0 |  |  |
| *Haplochromis sphex* | Critically endangered | | 0 |  |  |
| *Haplochromis sulphureus* | Critically endangered | | 0 |  |  |
| *Haplochromis teegelaari* | Critically endangered | | 0 |  |  |
| *Haplochromis teunisrasi* | Critically endangered | | 0 |  |  |
| *Haplochromis theliodon* | Critically endangered | | 0 |  |  |
| *Haplochromis ushindi* | Critically endangered | | 0 |  |  |
| *Haplochromis victorianus* | Critically endangered | | 0 |  |  |
| *Haplochromis vonlinnei* | Critically endangered | | 0 |  |  |
| *Haplochromis xenostoma* | Critically endangered | | 0 |  |  |
| *Hemibagrus punctatus* | Critically endangered | | 0 |  |  |
| *Heterotilapia cessiana* | Critically endangered | | 0 |  |  |
| *Hippoglossus hippoglossus* | Game fish | | 296 |  |  |
| *Hippoglossus stenolepis* | Game fish | | 57 |  |  |
| *Hoplias macrophthalmus* | Game fish | | 1 |  |  |
| *Horalabiosa arunachalami* | Critically endangered | | 0 |  |  |
| *Hubbsina turneri* | Critically endangered | | 0 |  |  |
| *Hucho bleekeri* | Critically endangered | | 2 |  |  |
| *Hucho hucho* | Game fish | | 12 |  |  |
| *Hucho taimen* | Game fish | | 26 |  |  |
| *Huso dauricus* | Critically endangered | | 9 |  |  |
| *Huso huso* | Critically endangered | | 133 |  |  |
| *Hydrocynus goliath* | Game fish | | 0 |  |  |
| *Hydrocynus vittatus* | Game fish | | 6 |  |  |
| *Hydrolycus scomberoides* | Game fish | | 0 |  |  |
| *Hypomesus transpacificus* | Critically endangered | | 37 |  |  |
| *Hypselobarbus pulchellus* | Critically endangered | | 0 |  |  |
| *Hypselobarbus thomassi* | Critically endangered | | 1 |  |  |
| *Iberochondrostoma almacai* | Critically endangered | | 0 |  |  |
| *Iberochondrostoma lusitanicum* | Both | | 3 |  |  |
| *Iberochondrostoma olisiponensis* | Critically endangered | | 2 |  |  |
| *Iberochondrostoma oretanum* | Critically endangered | | 0 |  |  |
| *Iberocypris palaciosi* | Critically endangered | | 0 |  |  |
| *Ictalurus furcatus* | Game fish | | 92 |  |  |
| *Ictalurus punctatus* | Game fish | | 1150 |  |  |
| *Ictiobus bubalus* | Game fish | | 2 |  |  |
| *Ictiobus cyprinellus* | Game fish | | 4 |  |  |
| *Ilyodon whitei* | Critically endangered | | 0 |  |  |
| *Isogomphodon oxyrhynchus* | Both | | 3 |  |  |
| *Istiompax indica* | Game fish | | 9 |  |  |
| *Istiophorus platypterus* | Game fish | | 21 |  |  |
| *Isurus oxyrinchus* | Both | | 49 |  |  |
| *Isurus paucus* | Game fish | | 4 |  |  |
| *Kajikia albida* | Game fish | | 15 |  |  |
| *Kajikia audax* | Game fish | | 30 |  |  |
| *Katsuwonus pelamis* | Game fish | | 111 |  |  |
| *Kiunga ballochi* | Critically endangered | | 0 |  |  |
| *Knipowitschia cameliae* | Critically endangered | | 0 |  |  |
| *Knipowitschia ephesi* | Critically endangered | | 0 |  |  |
| *Knipowitschia milleri* | Critically endangered | | 0 |  |  |
| *Knipowitschia mrakovcici* | Critically endangered | | 1 |  |  |
| *Knodus shinahota* | Critically endangered | | 0 |  |  |
| *Konia dikume* | Critically endangered | | 0 |  |  |
| *Konia eisentrauti* | Critically endangered | | 0 |  |  |
| *Kuhlia rupestris* | Game fish | | 2 |  |  |
| *Labeo curriei* | Critically endangered | | 0 |  |  |
| *Labeo mesops* | Critically endangered | | 0 |  |  |
| *Labeo porcellus* | Critically endangered | | 0 |  |  |
| *Labeo victorianus* | Critically endangered | | 8 |  |  |
| *Labeobarbus platystomus* | Critically endangered | | 0 |  |  |
| *Labeobarbus ruasae* | Critically endangered | | 0 |  |  |
| *Lamiopsis temminckii* | Game fish | | 1 |  |  |
| *Lamna nasus* | Both | | 15 |  |  |
| *Lamprologus kungweensis* | Critically endangered | | 0 |  |  |
| *Lateolabrax japonicus* | Game fish | | 95 |  |  |
| *Lateolabrax latus* | Game fish | | 1 |  |  |
| *Lates calcarifer* | Game fish | | 320 |  |  |
| *Lates niloticus* | Game fish | | 39 |  |  |
| *Latimeria chalumnae* | Critically endangered | | 8 |  |  |
| *Lepidomeda albivallis* | Critically endangered | | 1 |  |  |
| *Lepisosteus oculatus* | Game fish | | 14 |  |  |
| *Lepisosteus osseus* | Game fish | | 14 |  |  |
| *Lepisosteus platostomus* | Game fish | | 4 |  |  |
| *Lepisosteus platyrhincus* | Game fish | | 2 |  |  |
| *Lepomis auritus* | Game fish | | 7 |  |  |
| *Lepomis cyanellus* | Game fish | | 20 |  |  |
| *Lepomis gulosus* | Game fish | | 1 |  |  |
| *Lepomis macrochirus* | Game fish | | 270 |  |  |
| *Lepomis microlophus* | Game fish | | 10 |  |  |
| *Leucoraja circularis* | Critically endangered | | 0 |  |  |
| *Leucoraja fullonica* | Critically endangered | | 0 |  |  |
| *Leucoraja melitensis* | Critically endangered | | 0 |  |  |
| *Lichia amia* | Game fish | | 0 |  |  |
| *Lobotes surinamensis* | Game fish | | 10 |  |  |
| *Lota lota* | Game fish | | 111 |  |  |
| *Loxodon macrorhinus* | Game fish | | 1 |  |  |
| *Lucania interioris* | Critically endangered | | 1 |  |  |
| *Lucifuga simile* | Critically endangered | | 0 |  |  |
| *Luciobarbus brachycephalus* | Critically endangered | | 0 |  |  |
| *Luciobarbus subquincunciatus* | Critically endangered | | 0 |  |  |
| *Lutjanus analis* | Game fish | | 13 |  |  |
| *Lutjanus aratus* | Game fish | | 0 |  |  |
| *Lutjanus campechanus* | Game fish | | 167 |  |  |
| *Lutjanus cyanopterus* | Game fish | | 1 |  |  |
| *Lutjanus goldiei* | Game fish | | 0 |  |  |
| *Lutjanus novemfasciatus* | Game fish | | 0 |  |  |
| *Maccullochella peelii* | Both | | 62 |  |  |
| *Macquaria colonorum* | Game fish | | 23 |  |  |
| *Maculabatis arabica* | Critically endangered | | 0 |  |  |
| *Makaira nigricans* | Game fish | | 43 |  |  |
| *Maylandia usisyae* | Critically endangered | | 0 |  |  |
| *Mchenga conophoros* | Critically endangered | | 0 |  |  |
| *Megalops atlanticus* | Game fish | | 14 |  |  |
| *Melanochromis chipokae* | Critically endangered | | 0 |  |  |
| *Melanochromis lepidiadaptes* | Critically endangered | | 0 |  |  |
| *Menticirrhus undulatus* | Game fish | | 1 |  |  |
| *Mesonoemacheilus herrei* | Critically endangered | | 0 |  |  |
| *Metriaclima koningsi* | Critically endangered | | 0 |  |  |
| *Micropterus coosae* | Game fish | | 11 |  |  |
| *Micropterus dolomieu* | Game fish | | 190 |  |  |
| *Micropterus punctulatus* | Game fish | | 14 |  |  |
| *Micropterus salmoides* | Game fish | | 514 |  |  |
| *Moapa coriacea* | Critically endangered | | 1 |  |  |
| *Mogurnda furva* | Critically endangered | | 0 |  |  |
| *Mogurnda variegata* | Critically endangered | | 0 |  |  |
| *Morone americana* | Game fish | | 52 |  |  |
| *Morone chrysops* | Game fish | | 170 |  |  |
| *Morone mississippiensis* | Game fish | | 4 |  |  |
| *Morone saxatilis* | Game fish | | 649 |  |  |
| *Moxostoma anisurum* | Game fish | | 1 |  |  |
| *Moxostoma macrolepidotum* | Game fish | | 4 |  |  |
| *Mugilogobius amadi* | Critically endangered | | 0 |  |  |
| *Mustelus fasciatus* | Critically endangered | | 0 |  |  |
| *Myaka myaka* | Critically endangered | | 0 |  |  |
| *Mycteroperca microlepis* | Game fish | | 21 |  |  |
| *Nannostomus mortenthaleri* | Critically endangered | | 0 |  |  |
| *Narcine bancroftii* | Critically endangered | | 3 |  |  |
| *Nasolamia velox* | Game fish | | 0 |  |  |
| *Negaprion acutidens* | Game fish | | 1 |  |  |
| *Negaprion brevirostris* | Game fish | | 19 |  |  |
| *Nemacheilus troglocataractus* | Critically endangered | | 0 |  |  |
| *Nematistius pectoralis* | Game fish | | 2 |  |  |
| *Neochanna burrowsius* | Critically endangered | | 6 |  |  |
| *Neochanna heleios* | Critically endangered | | 0 |  |  |
| *Neolebias lozii* | Critically endangered | | 0 |  |  |
| *Neolebias powelli* | Critically endangered | | 0 |  |  |
| *Neolissochilus bovanicus* | Critically endangered | | 0 |  |  |
| *Notropis moralesi* | Critically endangered | | 0 |  |  |
| *Noturus crypticus* | Critically endangered | | 0 |  |  |
| *Nyassachromis breviceps* | Critically endangered | | 0 |  |  |
| *Ocyurus chrysurus* | Game fish | | 12 |  |  |
| *Odontaspis ferox* | Critically endangered | | 3 |  |  |
| *Oncorhynchus aguabonita* | Game fish | | 14 |  |  |
| *Oncorhynchus apache* | Both | | 6 |  |  |
| *Oncorhynchus clarkii* | Game fish | | 314 |  |  |
| *Oncorhynchus formosanus* | Critically endangered | | 1 |  |  |
| *Oncorhynchus gorbuscha* | Game fish | | 178 |  |  |
| *Oncorhynchus keta* | Game fish | | 291 |  |  |
| *Oncorhynchus kisutch* | Game fish | | 720 |  |  |
| *Oncorhynchus mykiss* | Game fish | | 4150 |  |  |
| *Oncorhynchus nerka* | Both | | 488 |  |  |
| *Oncorhynchus tshawytscha* | Game fish | | 1134 |  |  |
| *Ophiodon elongatus* | Game fish | | 27 |  |  |
| *Oplegnathus fasciatus* | Game fish | | 135 |  |  |
| *Oplegnathus punctatus* | Game fish | | 2 |  |  |
| *Orcynopsis unicolor* | Game fish | | 2 |  |  |
| *Oreochromis chungruruensis* | Critically endangered | | 0 |  |  |
| *Oreochromis esculentus* | Critically endangered | | 4 |  |  |
| *Oreochromis hunteri* | Critically endangered | | 0 |  |  |
| *Oreochromis jipe* | Critically endangered | | 0 |  |  |
| *Oreochromis karomo* | Critically endangered | | 0 |  |  |
| *Oreochromis karongae* | Critically endangered | | 6 |  |  |
| *Oreochromis lidole* | Critically endangered | | 3 |  |  |
| *Oreochromis mortimeri* | Both | | 1 |  |  |
| *Oreochromis squamipinnis* | Critically endangered | | 3 |  |  |
| *Oreochromis variabilis* | Critically endangered | | 0 |  |  |
| *Oreoglanis lepturus* | Critically endangered | | 0 |  |  |
| *Orthochromis uvinzae* | Critically endangered | | 0 |  |  |
| *Osteoglossum bicirrhosum* | Game fish | | 6 |  |  |
| *Oxynoemacheilus eregliensis* | Critically endangered | | 0 |  |  |
| *Oxynoemacheilus galilaeus* | Critically endangered | | 0 |  |  |
| *Oxynoemacheilus insignis* | Critically endangered | | 1 |  |  |
| *Oxynoemacheilus phoxinoides* | Critically endangered | | 0 |  |  |
| *Oxynoemacheilus seyhanensis* | Critically endangered | | 0 |  |  |
| *Oxynoemacheilus simavicus* | Critically endangered | | 0 |  |  |
| *Oxynoemacheilus theophilii* | Critically endangered | | 0 |  |  |
| *Oxynoemacheilus tigris* | Critically endangered | | 0 |  |  |
| *Oxynotus centrina* | Critically endangered | | 4 |  |  |
| *Pagrus auratus* | Game fish | | 163 |  |  |
| *Pagrus major* | Game fish | | 326 |  |  |
| *Pandaka pygmaea* | Critically endangered | | 0 |  |  |
| *Pangasianodon gigas* | Critically endangered | | 10 |  |  |
| *Pangasius sanitwongsei* | Critically endangered | | 0 |  |  |
| *Parabotia curtus* | Critically endangered | | 0 |  |  |
| *Parachanna africana* | Game fish | | 0 |  |  |
| *Parachanna insignis* | Game fish | | 0 |  |  |
| *Parachanna obscura* | Game fish | | 4 |  |  |
| *Parachondrostoma arrigonis* | Critically endangered | | 0 |  |  |
| *Paraclinus walkeri* | Critically endangered | | 0 |  |  |
| *Parahucho perryi* | Critically endangered | | 16 |  |  |
| *Paralabrax clathratus* | Game fish | | 9 |  |  |
| *Paralichthys californicus* | Game fish | | 38 |  |  |
| *Paralichthys dentatus* | Game fish | | 93 |  |  |
| *Parapsilorhynchus prateri* | Critically endangered | | 0 |  |  |
| *Paraschistura chrysicristinae* | Critically endangered | | 0 |  |  |
| *Paretroplus dambabe* | Critically endangered | | 0 |  |  |
| *Paretroplus gymnopreopercularis* | Critically endangered | | 0 |  |  |
| *Paretroplus maculatus* | Critically endangered | | 0 |  |  |
| *Paretroplus menarambo* | Critically endangered | | 0 |  |  |
| *Pelasgus epiroticus* | Critically endangered | | 0 |  |  |
| *Pelasgus laconicus* | Critically endangered | | 0 |  |  |
| *Pellona castelnaeana* | Game fish | | 0 |  |  |
| *Perca flavescens* | Game fish | | 311 |  |  |
| *Percina jenkinsi* | Critically endangered | | 1 |  |  |
| *Pethia bandula* | Critically endangered | | 0 |  |  |
| *Pethia pookodensis* | Critically endangered | | 1 |  |  |
| *Phoxinellus dalmaticus* | Critically endangered | | 0 |  |  |
| *Phractocephalus hemioliopterus* | Game fish | | 4 |  |  |
| *Phreatobius sanguijuela* | Critically endangered | | 1 |  |  |
| *Pimelodus grosskopfii* | Critically endangered | | 0 |  |  |
| *Plagopterus argentissimus* | Critically endangered | | 6 |  |  |
| *Poblana alchichica* | Critically endangered | | 0 |  |  |
| *Poecilia latipunctata* | Critically endangered | | 0 |  |  |
| *Poecilia sulphuraria* | Critically endangered | | 2 |  |  |
| *Pogonias cromis* | Game fish | | 18 |  |  |
| *Pollachius pollachius* | Game fish | | 12 |  |  |
| *Pollachius virens* | Game fish | | 77 |  |  |
| *Polydactylus macrochir* | Game fish | | 6 |  |  |
| *Polyprion americanus* | Both | | 6 |  |  |
| *Polysteganus undulosus* | Both | | 1 |  |  |
| *Pomatomus saltatrix* | Game fish | | 89 |  |  |
| *Pomatoschistus microps* | Critically endangered | | 16 |  |  |
| *Pomoxis annularis* | Game fish | | 42 |  |  |
| *Pomoxis nigromaculatus* | Game fish | | 45 |  |  |
| *Poropuntius chonglingchungi* | Critically endangered | | 0 |  |  |
| *Prionace glauca* | Both | | 79 |  |  |
| *Pristis pectinata* | Both | | 17 |  |  |
| *Pristis pristis* | Critically endangered | | 4 |  |  |
| *Pristis zijsron* | Both | | 4 |  |  |
| *Prosopium cylindraceum* | Game fish | | 9 |  |  |
| *Prosopium williamsoni* | Game fish | | 17 |  |  |
| *Proterorhinus tataricus* | Critically endangered | | 0 |  |  |
| *Psephurus gladius* | Critically endangered | | 3 |  |  |
| *Pseudobagrus medianalis* | Critically endangered | | 0 |  |  |
| *Pseudobarbus burchelli* | Critically endangered | | 1 |  |  |
| *Pseudobarbus erubescens* | Critically endangered | | 0 |  |  |
| *Pseudobarbus senticeps* | Critically endangered | | 0 |  |  |
| *Pseudobatos horkelii* | Critically endangered | | 1 |  |  |
| *Pseudophoxinus atropatenus* | Critically endangered | | 0 |  |  |
| *Pseudophoxinus elizavetae* | Critically endangered | | 0 |  |  |
| *Pseudophoxinus hasani* | Critically endangered | | 0 |  |  |
| *Pseudophoxinus maeandricus* | Critically endangered | | 0 |  |  |
| *Pseudophoxinus ninae* | Critically endangered | | 0 |  |  |
| *Pseudophoxinus sojuchbulagi* | Critically endangered | | 0 |  |  |
| *Pseudophoxinus syriacus* | Critically endangered | | 0 |  |  |
| *Pseudoplatystoma corruscans* | Game fish | | 25 |  |  |
| *Pseudoplatystoma fasciatum* | Game fish | | 24 |  |  |
| *Pseudoplatystoma reticulatum* | Game fish | | 21 |  |  |
| *Pseudoplatystoma tigrinum* | Game fish | | 0 |  |  |
| *Pseudoscaphirhynchus fedtschenkoi* | Critically endangered | | 0 |  |  |
| *Pseudoscaphirhynchus hermanni* | Critically endangered | | 0 |  |  |
| *Pseudoscaphirhynchus kaufmanni* | Critically endangered | | 1 |  |  |
| *Pseudotropheus cyaneorhabdos* | Critically endangered | | 0 |  |  |
| *Psilorhynchus tenura* | Critically endangered | | 0 |  |  |
| *Ptychidio jordani* | Critically endangered | | 1 |  |  |
| *Ptychochromis insolitus* | Critically endangered | | 0 |  |  |
| *Ptychochromoides betsileanus* | Critically endangered | | 0 |  |  |
| *Ptychochromoides itasy* | Critically endangered | | 0 |  |  |
| *Pungitius hellenicus* | Critically endangered | | 0 |  |  |
| *Pungu maclareni* | Critically endangered | | 0 |  |  |
| *Puntius deccanensis* | Critically endangered | | 0 |  |  |
| *Pygocentrus nattereri* | Game fish | | 6 |  |  |
| *Pylodictis olivaris* | Game fish | | 32 |  |  |
| *Rachycentron canadum* | Game fish | | 196 |  |  |
| *Rhabdalestes leleupi* | Critically endangered | | 0 |  |  |
| *Rhamdella montana* | Critically endangered | | 0 |  |  |
| *Rhamdia xetequepeque* | Critically endangered | | 0 |  |  |
| *Rheocles derhami* | Critically endangered | | 0 |  |  |
| *Rheocles lateralis* | Critically endangered | | 0 |  |  |
| *Rhizoprionodon acutus* | Game fish | | 4 |  |  |
| *Rhizoprionodon lalandii* | Game fish | | 6 |  |  |
| *Rhizoprionodon longurio* | Game fish | | 0 |  |  |
| *Rhizoprionodon oligolinx* | Game fish | | 2 |  |  |
| *Rhizoprionodon porosus* | Game fish | | 3 |  |  |
| *Rhizoprionodon taylori* | Game fish | | 3 |  |  |
| *Rhizoprionodon terraenovae* | Game fish | | 15 |  |  |
| *Rhizosomichthys totae* | Critically endangered | | 0 |  |  |
| *Romanichthys valsanicola* | Critically endangered | | 0 |  |  |
| *Rostroraja alba* | Both | | 1 |  |  |
| *Salaria economidisi* | Critically endangered | | 0 |  |  |
| *Salmo carpio* | Critically endangered | | 1 |  |  |
| *Salmo ezenami* | Critically endangered | | 0 |  |  |
| *Salmo platycephalus* | Critically endangered | | 2 |  |  |
| *Salmo salar* | Game fish | | 4016 |  |  |
| *Salmo trutta* | Game fish | | 1304 |  |  |
| *Salvelinus alpinus* | Game fish | | 531 |  |  |
| *Salvelinus confluentus* | Game fish | | 132 |  |  |
| *Salvelinus fontinalis* | Game fish | | 553 |  |  |
| *Salvelinus grayi* | Critically endangered | | 0 |  |  |
| *Salvelinus lonsdalii* | Critically endangered | | 0 |  |  |
| *Salvelinus malma* | Game fish | | 48 |  |  |
| *Salvelinus namaycush* | Game fish | | 317 |  |  |
| *Salvelinus obtusus* | Critically endangered | | 0 |  |  |
| *Sander canadensis* | Game fish | | 29 |  |  |
| *Sander lucioperca* | Game fish | | 167 |  |  |
| *Sander vitreus* | Game fish | | 557 |  |  |
| *Sarda australis* | Game fish | | 2 |  |  |
| *Sarda chiliensis* | Game fish | | 1 |  |  |
| *Sarda lineolata* | Game fish | | 2 |  |  |
| *Sarda orientalis* | Game fish | | 3 |  |  |
| *Sarda sarda* | Game fish | | 11 |  |  |
| *Sarotherodon caroli* | Critically endangered | | 0 |  |  |
| *Sarotherodon linnellii* | Critically endangered | | 0 |  |  |
| *Sarotherodon lohbergeri* | Critically endangered | | 0 |  |  |
| *Sarotherodon steinbachi* | Critically endangered | | 0 |  |  |
| *Scaphirhynchus suttkusi* | Critically endangered | | 2 |  |  |
| *Scaphognathops theunensis* | Critically endangered | | 0 |  |  |
| *Scardinius graecus* | Critically endangered | | 0 |  |  |
| *Scardinius racovitzai* | Critically endangered | | 0 |  |  |
| *Scardinius scardafa* | Critically endangered | | 0 |  |  |
| *Scaturiginichthys vermeilipinnis* | Critically endangered | | 1 |  |  |
| *Schistura leukensis* | Critically endangered | | 0 |  |  |
| *Schistura nasifilis* | Critically endangered | | 0 |  |  |
| *Schistura papulifera* | Critically endangered | | 0 |  |  |
| *Schistura spiloptera* | Critically endangered | | 0 |  |  |
| *Schistura tenura* | Critically endangered | | 0 |  |  |
| *Schizothorax grahami* | Critically endangered | | 0 |  |  |
| *Schizothorax integrilabiatus* | Critically endangered | | 0 |  |  |
| *Schizothorax nepalensis* | Critically endangered | | 0 |  |  |
| *Schizothorax raraensis* | Critically endangered | | 0 |  |  |
| *Sciaena callaensis* | Critically endangered | | 0 |  |  |
| *Sciaenops ocellatus* | Game fish | | 228 |  |  |
| *Scoliodon laticaudus* | Game fish | | 4 |  |  |
| *Scomberoides commersonnianus* | Game fish | | 3 |  |  |
| *Scomberoides lysan* | Game fish | | 0 |  |  |
| *Scomberomorus cavalla* | Game fish | | 25 |  |  |
| *Scomberomorus commerson* | Game fish | | 31 |  |  |
| *Scomberomorus maculatus* | Game fish | | 11 |  |  |
| *Scomberomorus regalis* | Game fish | | 0 |  |  |
| *Scomberomorus sierra* | Game fish | | 2 |  |  |
| *Scriptaphyosemion cauveti* | Critically endangered | | 0 |  |  |
| *Scriptaphyosemion etzeli* | Critically endangered | | 0 |  |  |
| *Sebastes melanops* | Game fish | | 77 |  |  |
| *Sebastes mystinus* | Game fish | | 11 |  |  |
| *Sebastes paucispinis* | Both | | 11 |  |  |
| *Sebastes ruberrimus* | Game fish | | 10 |  |  |
| *Seriola dumerili* | Game fish | | 100 |  |  |
| *Seriola lalandi* | Game fish | | 111 |  |  |
| *Seriola rivoliana* | Game fish | | 15 |  |  |
| *Serranochromis robustus* | Both | | 1 |  |  |
| *Sewellia albisuera* | Critically endangered | | 0 |  |  |
| *Sewellia breviventralis* | Critically endangered | | 0 |  |  |
| *Silurus glanis* | Game fish | | 59 |  |  |
| *Silurus mento* | Critically endangered | | 0 |  |  |
| *Sinocyclocheilus grahami* | Critically endangered | | 3 |  |  |
| *Sinocyclocheilus yangzongensis* | Critically endangered | | 0 |  |  |
| *Speoplatyrhinus poulsoni* | Critically endangered | | 0 |  |  |
| *Sphaerophysa dianchiensis* | Critically endangered | | 0 |  |  |
| *Sphyraena afra* | Game fish | | 0 |  |  |
| *Sphyraena barracuda* | Game fish | | 7 |  |  |
| *Sphyrna corona* | Game fish | | 0 |  |  |
| *Sphyrna couardi* | Game fish | | 0 |  |  |
| *Sphyrna lewini* | Game fish | | 13 |  |  |
| *Sphyrna media* | Game fish | | 0 |  |  |
| *Sphyrna mokarran* | Game fish | | 5 |  |  |
| *Sphyrna tiburo* | Game fish | | 13 |  |  |
| *Sphyrna tudes* | Game fish | | 0 |  |  |
| *Sphyrna zygaena* | Both | | 4 |  |  |
| *Squalius cappadocicus* | Critically endangered | | 0 |  |  |
| *Squalus acanthias* | Both | | 67 |  |  |
| *Squatina aculeata* | Critically endangered | | 1 |  |  |
| *Squatina oculata* | Critically endangered | | 0 |  |  |
| *Squatina squatina* | Both | | 5 |  |  |
| *Stenodus leucichthys* | Game fish | | 6 |  |  |
| *Stereolepis gigas* | Both | | 5 |  |  |
| *Stiphodon discotorquatus* | Critically endangered | | 0 |  |  |
| *Stiphodon rubromaculatus* | Critically endangered | | 0 |  |  |
| *Stomatepia mariae* | Critically endangered | | 0 |  |  |
| *Stomatepia mongo* | Critically endangered | | 0 |  |  |
| *Stomatepia pindu* | Critically endangered | | 0 |  |  |
| *Sturisomatichthys frenatum* | Critically endangered | | 0 |  |  |
| *Syngnathus watermeyeri* | Critically endangered | | 0 |  |  |
| *Synodontis dekimpei* | Critically endangered | | 0 |  |  |
| *Systomus compressiformis* | Critically endangered | | 0 |  |  |
| *Takifugu chinensis* | Critically endangered | | 1 |  |  |
| *Tampichthys mandibularis* | Critically endangered | | 0 |  |  |
| *Tautoga onitis* | Game fish | | 28 |  |  |
| *Teleogramma brichardi* | Critically endangered | | 0 |  |  |
| *Telestes fontinalis* | Critically endangered | | 0 |  |  |
| *Telestes polylepis* | Critically endangered | | 0 |  |  |
| *Telestes turskyi* | Critically endangered | | 0 |  |  |
| *Tetrapleurodon spadiceus* | Critically endangered | | 2 |  |  |
| *Tetrapturus angustirostris* | Game fish | | 0 |  |  |
| *Tetrapturus belone* | Game fish | | 0 |  |  |
| *Tetrapturus pfluegeri* | Game fish | | 1 |  |  |
| *Thunnus alalunga* | Game fish | | 87 |  |  |
| *Thunnus albacares* | Game fish | | 122 |  |  |
| *Thunnus atlanticus* | Game fish | | 6 |  |  |
| *Thunnus maccoyii* | Both | | 84 |  |  |
| *Thunnus obesus* | Game fish | | 69 |  |  |
| *Thunnus thynnus* | Game fish | | 213 |  |  |
| *Thunnus tonggol* | Game fish | | 10 |  |  |
| *Thymallus arcticus* | Game fish | | 46 |  |  |
| *Thymallus thymallus* | Game fish | | 75 |  |  |
| *Tilapia bakossiorum* | Critically endangered | | 0 |  |  |
| *Tilapia bemini* | Critically endangered | | 0 |  |  |
| *Tilapia bythobates* | Critically endangered | | 0 |  |  |
| *Tilapia deckerti* | Critically endangered | | 0 |  |  |
| *Tilapia flava* | Critically endangered | | 0 |  |  |
| *Tilapia guinasana* | Critically endangered | | 0 |  |  |
| *Tilapia gutturosa* | Critically endangered | | 0 |  |  |
| *Tilapia imbriferna* | Critically endangered | | 0 |  |  |
| *Tilapia snyderae* | Critically endangered | | 0 |  |  |
| *Tilapia spongotroktis* | Critically endangered | | 0 |  |  |
| *Tilapia thysi* | Critically endangered | | 0 |  |  |
| *Tinca tinca* | Game fish | | 155 |  |  |
| *Tor remadevii* | Critically endangered | | 1 |  |  |
| *Torpedo suessii* | Critically endangered | | 0 |  |  |
| *Totoaba macdonaldi* | Both | | 25 |  |  |
| *Trachinotus falcatus* | Game fish | | 5 |  |  |
| *Triaenodon obesus* | Game fish | | 5 |  |  |
| *Trichomycterus venulosus* | Critically endangered | | 0 |  |  |
| *Trigonostigma somphongsi* | Critically endangered | | 0 |  |  |
| *Typhleotris mararybe* | Critically endangered | | 0 |  |  |
| *Typhleotris pauliani* | Critically endangered | | 0 |  |  |
| *Urogymnus polylepis* | Critically endangered | | 0 |  |  |
| *Urolophus javanicus* | Critically endangered | | 0 |  |  |
| *Valencia hispanica* | Critically endangered | | 0 |  |  |
| *Valencia letourneuxi* | Critically endangered | | 1 |  |  |
| *Xenoclarias eupogon* | Critically endangered | | 0 |  |  |
| *Xenocypris yunnanensis* | Critically endangered | | 0 |  |  |
| *Xiphias gladius* | Game fish | | 97 |  |  |
| *Xiphophorus couchianus* | Critically endangered | | 0 |  |  |
| *Xyrauchen texanus* | Critically endangered | | 41 |  |  |
| *Yunnanilus discoloris* | Critically endangered | | 0 |  |  |
| *Zingel asper* | Critically endangered | | 6 |  |  |
| *Zoogoneticus tequila* | Critically endangered | | 1 |  |  |

Table S2. Total number of articles by journal with the research subject of the article including one of the game fishes or critically endangered fishes identified by the International Game Fish Association or the International Union for Conservation of Nature Red List, respectively. Articles were identified using specific search criteria in Web of Science search engine in 2019. The first year of data reflects the first year for which the journal is included in the Web of Science database, not the first year of journal publication; max=maximum number of articles in a year with the research subject including one of the game fishes or critically endangered fishes identified by the International Game Fish Association or the International Union for Conservation of Nature Red List, respectively; min = minimum number of articles in a year with the research subject including one of the game fishes or critically endangered fishes identified by the International Game Fish Association or the International Union for Conservation of Nature Red List, respectively.

| Journal | First year of data | Years of data collected | Mean | Median | Max | Min | All articles from first year of data |
| --- | --- | --- | --- | --- | --- | --- | --- |
| Acta Ichthyolodica et Piscatoria* |  |  |  |  |  |  |  |
| American Midland Naturalist | 1966 | 28 | 2 | 1 | 4 | 1 | 45 |
| American Museum Novitates | 2007 | 1 | 1 | 1 | 1 | 1 | 1 |
| Animal Biodiversity and Conservation | 2011 | 3 | 1 | 1 | 1 | 1 | 3 |
| Animal Conservation | 2001 | 8 | 1 | 1 | 2 | 1 | 9 |
| Aquacultural Engineering | 1986 | 33 | 5 | 4 | 13 | 1 | 163 |
| Aquaculture | 1974 | 45 | 100 | 104 | 220 | 6 | 4512 |
| Aquaculture Economics & Management | 2012 | 6 | 2 | 3 | 4 | 1 | 14 |
| Aquaculture Environment Interactions | 2010 | 9 | 7 | 8 | 12 | 1 | 63 |
| Aquaculture International | 1996 | 23 | 14 | 11 | 34 | 3 | 332 |
| Aquaculture Nutrition | 1995 | 24 | 29 | 22 | 67 | 14 | 687 |
| Aquaculture Reports | 2015 | 4 | 7 | 8 | 12 | 2 | 29 |
| Aquaculture Research | 1997 | 22 | 58 | 55 | 136 | 29 | 1280 |
| Aquatic Living Resources | 1988 | 29 | 7 | 7 | 16 | 1 | 214 |
| Avian Conservation and Ecology* |  |  |  |  |  |  |  |
| Biodiversity and Conservation | 2010 | 2 | 2 | 2 | 2 | 1 | 3 |
| Biodiversity Data Journal | 2017 | 1 | 1 | 1 | 1 | 1 | 1 |
| Bioinvasions Records | 2012 | 4 | 2 | 2 | 4 | 1 | 9 |
| Biological Conservation | 1977 | 23 | 2 | 2 | 6 | 1 | 51 |
| Biological Invasions | 2006 | 12 | 4 | 4 | 9 | 1 | 43 |
| Biota Neotropica | 2011 | 2 | 1 | 1 | 1 | 1 | 2 |
| Bird Conservation International* |  |  |  |  |  |  |  |
| Boletim do Instituto de Pesca | 2008 | 11 | 6 | 7 | 12 | 1 | 70 |
| Bulletin of the European Association of Fish Pathologists* |  |  |  |  |  |  |  |
| Bulletin of the American Museum of Natural History* |  |  |  |  |  |  |  |
| Bulletin of the Peabody Museum of Natural History * |  |  |  |  |  |  |  |
| California Cooperative Oceanic Fisheries Investigations Reports | 1988 | 21 | 1 | 1 | 3 | 1 | 28 |
| California Cooperative Oceanic Fisheries Investigations Reports Fishery Bulletin* |  |  |  |  |  |  |  |
| California Fish and Game | 1966 | 52 | 4 | 3 | 9 | 1 | 183 |
| Canadian Journal of Fisheries and Aquatic Sciences | 1980 | 39 | 64 | 59 | 108 | 36 | 2492 |
| CCAMLR Science* |  |  |  |  |  |  |  |
| Conservation Biology | 1992 | 20 | 2 | 2 | 5 | 1 | 41 |
| Conservation Genetics | 2001 | 18 | 9 | 9 | 17 | 2 | 157 |
| Conservation Genetics Resources | 2009 | 10 | 10 | 9 | 16 | 3 | 99 |
| Conservation Letters | 2009 | 1 | 1 | 1 | 1 | 1 | 1 |
| Conservation Physiology | 2013 | 6 | 7 | 6 | 10 | 4 | 39 |
| Developmental and Comparative Immunology | 1980 | 39 | 14 | 11 | 40 | 3 | 540 |
| Diseases of Aquatic Organisms | 1986 | 33 | 24 | 26 | 45 | 7 | 807 |
| Diversity and Distributions | 2009 | 4 | 2 | 1 | 3 | 1 | 6 |
| Eco Mont-journal on Protected Mountain Areas Research | 2015 | 1 | 1 | 1 | 1 | 1 | 1 |
| Ecography | 1992 | 7 | 1 | 1 | 1 | 1 | 7 |
| Ecology of Freshwater Fish | 1997 | 22 | 21 | 23 | 37 | 9 | 455 |
| Endangered Species Research | 2012 | 6 | 5 | 4 | 10 | 1 | 29 |
| Environmental Conservation | 2013 | 1 | 1 | 1 | 1 | 1 | 1 |
| Fish & Shellfish Immunology | 1993 | 26 | 54 | 43 | 125 | 10 | 1409 |
| Fish and Fisheries | 2000 | 6 | 2 | 2 | 4 | 1 | 12 |
| Fish Pathology | 1986 | 33 | 5 | 5 | 11 | 1 | 152 |
| Fish Physiology and Biochemistry | 1986 | 33 | 33 | 32 | 73 | 12 | 1093 |
| Fisheries | 1981 | 34 | 2 | 2 | 7 | 1 | 84 |
| Fisheries Management and Ecology | 1999 | 20 | 16.5 | 16 | 26 | 7 | 330 |
| Fisheries Oceanography | 1995 | 24 | 8 | 7 | 15 | 2 | 188 |
| Fisheries Research | 1982 | 36 | 21 | 18 | 58 | 1 | 756 |
| Fisheries Science | 1994 | 25 | 21 | 22 | 35 | 9 | 525 |
| Fishery Bulletin | 1972 | 47 | 13 | 11 | 27 | 2 | 588 |
| Global Change Biology | 2006 | 11 | 3 | 3 | 4 | 1 | 30 |
| Global Ecology and Conservation | 2014 | 4 | 2 | 2 | 2 | 2 | 8 |
| Human Dimensions of Wildlife* |  |  |  |  |  |  |  |
| Human-Wildlife Interactions* |  |  |  |  |  |  |  |
| Ices Journal of Marine Science | 1991 | 28 | 18 | 16 | 54 | 2 | 517 |
| Indian Journal of Fisheries | 2009 | 10 | 9 | 9 | 18 | 2 | 92 |
| Iranian Journal of Fisheries Sciences | 2007 | 12 | 19 | 22 | 30 | 3 | 223 |
| Israeli Journal of Aquaculture-Bamidgeh | 1988 | 31 | 8 | 7 | 19 | 1 | 243 |
| Journal for Nature Conservation* |  |  |  |  |  |  |  |
| Journal of Applied Ecology | 1967 | 26 | 1 | 1 | 3 | 1 | 35 |
| Journal of Applied Ichthyology | 1991 | 28 | 28 | 22 | 107 | 1 | 793 |
| Journal of Aquatic Animal Health | 1997 | 22 | 15 | 14 | 28 | 7 | 326 |
| Journal of Ethnobiology and Ethnomedicine | 2014 | 1 | 1 | 1 | 1 | 1 | 1 |
| Journal of Fish and Wildlife Management | 2010 | 9 | 5 | 6 | 11 | 2 | 48 |
| Journal of Fish Biology | 1969 | 50 | 64 | 65 | 114 | 8 | 3196 |
| Journal of Fish Diseases | 1978 | 41 | 29 | 25 | 58 | 11 | 1189 |
| Journal of Natural History | 1972 | 8 | 1 | 1 | 2 | 1 | 10 |
| Journal of the World Aquaculture Society | 1996 | 23 | 23 | 24 | 33 | 17 | 532 |
| Knowledge and Management of Aquatic Ecosystems | 2008 | 11 | 3 | 4 | 6 | 1 | 37 |
| Koedoe | 2018 | 1 | 1 | 1 | 1 | 1 | 1 |
| Landscape and Ecological Engineering* |  |  |  |  |  |  |  |
| Latin America Journal of Aquatic Research* |  |  |  |  |  |  |  |
| Management of Biological Invasions | 2017 | 2 | 4 | 4 | 6 | 2 | 8 |
| Marine and Coastal Fisheries | 2009 | 10 | 12 | 11 | 24 | 5 | 115 |
| Marine and Freshwater Research | 1995 | 24 | 11 | 9 | 24 | 4 | 255 |
| Marine Biodiversity | 2016 | 3 | 3 | 2 | 5 | 1 | 8 |
| Marine Resource Economics | 2008 | 6 | 2 | 1 | 3 | 1 | 9 |
| Natural History | 1973 | 6 | 1 | 1 | 1 | 1 | 6 |
| Nature Conservation-Bulgaria* |  |  |  |  |  |  |  |
| Natureza & Conservção | 2008 | 3 | 1 | 1 | 1 | 1 | 3 |
| NeoBiota* |  |  |  |  |  |  |  |
| New Zealand Journal of Marine and Freshwater Research | 1977 | 39 | 3 | 3 | 9 | 1 | 129 |
| Nippon Suisan Gakkaishi | 1987 | 32 | 12 | 10 | 29 | 5 | 384 |
| North American Journal of Aquaculture | 1999 | 20 | 29 | 28 | 44 | 13 | 572 |
| North American Journal of Fisheries Management | 2001 | 18 | 59 | 56 | 84 | 42 | 1060 |
| Northeastern Naturalist | 2001 | 11 | 3 | 3 | 5 | 1 | 28 |
| Oryx | 2006 | 2 | 1 | 1 | 1 | 1 | 2 |
| Pachyderm* |  |  |  |  |  |  |  |
| Palaeobiodiversity and Palaeoenvironments* |  |  |  |  |  |  |  |
| Paleobiology* |  |  |  |  |  |  |  |
| Perspectives in Ecology and Conservation* |  |  |  |  |  |  |  |
| Polar Biology | 1987 | 16 | 2 | 2 | 4 | 1 | 32 |
| Proceedings of the Academy of Natural Sciences of Philadelphia | 2010 | 1 | 1 | 1 | 1 | 1 | 1 |
| Proceedings of the Linnean Society of New South Wales | 2015 | 1 | 1 | 1 | 1 | 1 | 1 |
| Reviews in Aquaculture | 2009 | 3 | 1 | 1 | 1 | 1 | 3 |
| Reviews in Fish Biology and Fisheries | 1995 | 13 | 4 | 2 | 19 | 1 | 46 |
| Reviews in Fisheries Science & Aquaculture | 2014 | 4 | 2 | 2 | 4 | 1 | 9 |
| Revista Chilena de Historia Natural | 1990 | 3 | 1 | 1 | 1 | 1 | 3 |
| Revista Mexicana de Biodiversidad | 2013 | 5 | 1 | 1 | 2 | 1 | 7 |
| Southeastern Naturalist | 2002 | 15 | 2 | 1 | 6 | 1 | 32 |
| Southwestern Naturalist | 1982 | 29 | 2 | 2 | 3 | 1 | 50 |
| Systematics and Biodiversity | 2005 | 1 | 1 | 1 | 1 | 1 | 1 |
| Transactions of the American Fisheries Society | 1965 | 54 | 45 | 45 | 80 | 14 | 2442 |
| Tropical Conservation Science | 2013 | 1 | 1 | 1 | 1 | 1 | 1 |
| Turkish Journal of Fisheries and Aquatic Sciences | 2008 | 11 | 21 | 24 | 33 | 7 | 231 |
| Urban Ecosystems | 2018 | 1 | 2 | 2 | 2 | 2 | 2 |
| Western North American Naturalist | 2000 | 14 | 2 | 2 | 6 | 1 | 32 |
| Wildlife Society Bulletin* |  |  |  |  |  |  |  |

*Data not available because the journal had zero articles for the species searched.

## Supplemental Figures


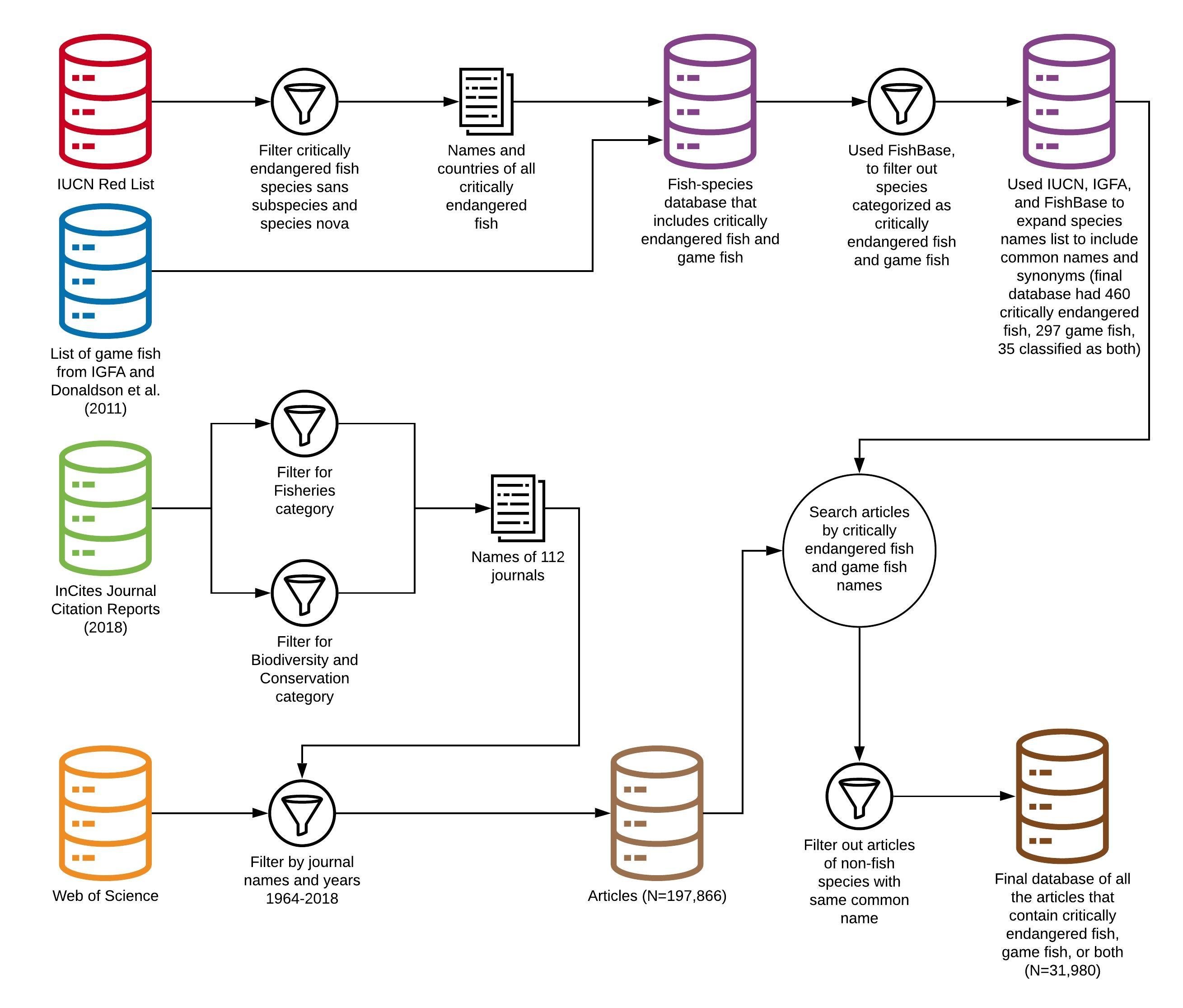


fig. S1. Flowchart illustrating databases and filters used to create a final database that contained 31,980 articles where names of critically endangered fishes, game fishes, or both were included in the title of the article. The abbreviations IUCN is for International Union for Conservation of Nature and IGFA is for the International Game Fish Association. Figure was created using freely available Lucidchart (URL: www.lucidchart.com).
